# Real-Space Constrained Density Functional Theory Investigation of Site-Specific, Interfacial Charge Recombination Dynamics Across the Au Nanoparticle/TiO_2_ Heterojunction

**DOI:** 10.1021/acs.jpclett.5c02905

**Published:** 2026-01-06

**Authors:** Drew M. Glenna, Carlos Mora Perez, Ernest Hermosillo, Haiyan Zhao, Jin Qian

**Affiliations:** † Department of Nuclear Engineering & Industrial Management, Center for Advanced Energy Studies, 5640 University of Idaho, Idaho Falls, Idaho 83402, United States; ‡ Chemical Sciences Division, 1666 Lawrence Berkeley National Laboratory, Berkeley, California 94720, United States; § Department of Chemical and Biological Engineering, Center for Advanced Energy Studies, 5640 University of Idaho, Idaho Falls, Idaho 83402, United States

## Abstract

Au nanoparticle (NP)/TiO_2_ heterojunction is a representative system to study interfacial charge transfer in photocatalysis and photovoltaics, where suppressing recombination from TiO_2_ to Au can enhance hot carrier extraction. We apply real-space constrained density functional theory (CDFT) with Marcus theory to quantify charge recombination time scales across Au/TiO_2_. This approach enables direct control and visualization of charge-separated states, aligning with site-specific probes like time-resolved X-ray photoelectron spectroscopy (trXPS). We find that the charge-separated state features a bipolaron, with recombination dominated by TiO_2_ LUMO to Au HOMO transitions, primarily at interfacial Au sites. Marcus rate predictions are benchmarked with surface hopping methods, quantifying differences in time scales and computational efficiency. Lastly, we examine how the Au cluster size affects the free energy change (Δ*G*) and reorganization energy (λ), explaining trends in closed-shell systems and highlighting challenges for open-shell extrapolations. Overall, CDFT + Marcus theory provides efficient, mechanistically transparent interfacial charge transfer modeling, and we clearly defined its applicability and limitation.

## Graphical abstract

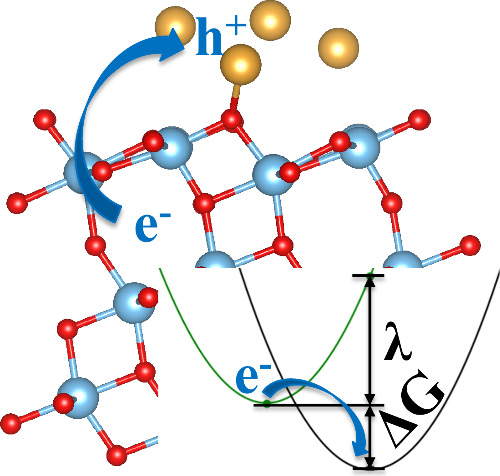

Metal NPs efficiently capture light through plasmonic enhancement, subsequently dephasing into hot electrons and holes that can either drive chemical reactions^1–5^ or transfer across metal-SC interfaces enabling promising photocatalytic and photovoltaic applications.^6–20^ Among the noble metals, Au stands out due to its superior chemical stability compared to silver, copper, and aluminum, which are easily oxidized at the nanoscale. The surface plasmon resonance of Au NPs lies within the visible light range (e.g., ∼532 nm for Au NPs), and the ideal band alignment between Au NPs and TiO_2_ substrates renders the Au/TiO_2_ interface an attractive model system for both theoretical and experimental investigations.^1,11,16,18,21^ The overall efficiency of a plasmonic hot-carrier device depends on three key factors: the activated energy distribution of the hot carrier, its transport through the metal, and most critically its transport across the metal-SC interface.^16^ Understanding the charge transfer bottleneck, mechanism, and associated time scales, all of which presumably closely depend on the NP size/shape effect, represents a crucial step toward futuristic renewable energy technologies.

Early first-principles plasmonic NPs research relied predominantly on time-dependent DFT (including linear-response TDDFT and real-space TDDFT) that successfully explained a range of optical responses of small metal clusters and their dimers.^22–25^ However, the grand renewable energy vision of harvesting these plasmonically generated hot carriers has shifted the focus toward understanding the plasmon decay mechanisms and hot carrier dynamics.^11,13,14,16–19,21^ Recent studies have contributed to the knowledge of these recombination processes by leveraging nonadiabatic molecular dynamics (NAMD) techniques,^18^ specialized plane-wave Kohn–Sham (KS) DFT methods (e.g., JDFTx,^16,26^ PWmat,^27^ and PEtot^20^ (combined with GW and Marcus theory^28^)), and customized wave function embedded DFT methods.^1^ Holistically, these computational studies described a mechanistic picture in which the plasmon–derived electron–hole pair in the Au NP follows one of two fates: (i) intraparticle thermalization, or (ii) hot–electron transfer into the TiO_2_ SC. The latter route generates a fleeting charge-separated excited state; the injected electron undergoes phonon-assisted back-transfer to recombine with the residual hole in the Au NP, thereby returning to the system’s ground-state. The longer lifetime for this fleeting charge-separated excited state, the more possibilities that we can harvest the hot carriers for chemical reactions. Here, surface hopping algorithms within NAMD are the most established for predicting charge transfer dynamics across the Au/TiO_2_ heterojunction.^18^ The fewest-switches surface hopping (FSSH^29^) technique successfully reproduces the sub-240 fs (fs) charge injection time scale, while the decoherence-induced surface hopping (DISH^30^) extension is in sync with the picosecond (ps) charge recombination time scale documented by time-resolved X-ray Photoelectron Spectroscopy (trXPS).^18^

An appealing complementary strategy to the established NAMD simulations is real-space constrained DFT (CDFT).^31^ It is computationally less intensive, and site-specific in its buildup, which in principle, reflects the physical picture measured by trXPS experiment.^21^ CDFT allows for the direct generation of diabatic electronic states by localizing (or delocalizing) charge on specific atoms (or fragments of atoms) and accurately describes charge-separated excited states. By staying within the broader framework of DFT, one can leverage advanced functionals to partially mitigate self-interaction error (SIE), to capture static correlation effects, while retaining DFT-level computational efficiency. By coupling the energetics from CDFT calculations (Δ*G*, λ, and the diabatic coupling term *H*_ab_) with Marcus theory, one can also obtain ultrafast charge transfer time scales. The CDFT+Marcus theory approach has been successfully applied to molecular,^31^ solid-electrolyte interface,^31–33^ and polaron hopping in condensed phase^34–36^ systems. However, extending this theoretical framework to interfacial condensed matter systems such as Au/TiO_2_ remains nontrivial due to the highly delocalized electronic states and difficulty defining nuclear coordinates at such interfaces.

In this context, we systematically assess the applicability, advantages, and limitations of CDFT + Marcus theory to study site-specific charge transfer phenomena across the Au/TiO_2_ heterojunction. We focus on charge recombination, pinpoint the predominant recombination channels and the most active Au sites via electronic structure and charge-partitioning analyses, respectively. The most suitable Marcus rate expression is identified and compared with NAMD lifetimes (FSSH/DISH). Within current CDFT capabilities, we further examine how charge recombination dynamics vary with Au cluster size.

More precisely, we investigate charge recombination across a 4Au atom cluster on a 3-layer anatase TiO_2_ (101) slab (4Au/3TiO_2_). Ab-initio molecular dynamics (AIMD) are performed with the Vienna Ab initio Simulation Package (VASP^37–40^ ), and real-space DFT/CDFT calculations with the Grid-Based Projector-Augmented Waves (GPAW^41–44^ ) software. DFT yields charge-recombined ground-state energies, whereas CDFT provides charge-separated excited-state energetics, serving as inputs for Marcus theory to predict recombination time scales. Additional computational details like the Au/TiO_2_ cell geometry, DFT and CDFT calculation set ups, procedure for predicting charge recombination time scales, NAMD techniques, and calibrating CDFT constraints are given in Sections S1–S3. For completeness of the study, limitations of the current methods are also discussed in Sections S4 and S5.

In CDFT, excited-state energetics are approximated by supplying a Lagrange multiplier term to the KS-DFT energy functional (*E*[ρ­(*r*)]),

1 $$E\left[N_{c}\right]=\frac{\min}{\rho }\frac{\max}{V_{c}}\left[E\left[\rho \left(r\right)\right]+\underset{c}{\sum }V_{c}\left(\int w_{c}\left(r\right)\rho \left(r\right)d^{3}r-N_{c}\right)\right]$$

where *V*_c_ is the constraining potential, *N*_c_ is the constraining charge, and *∫w*_c_(*r*)­ρ­(*r*)*d* ^3^ *r* is the volume integral over the electron density (ρ) of a fragment of atoms.^31^ GPAW adopts Hirshfeld-type charge partitioning for the weight function *w*_c_(*r*), which is determined by

2a $$w_{c}\left(r\right)=\frac{\underset{j\in c}{\sum }\rho_{j}\left(r\right)}{\underset{k}{\sum }\rho_{k}\left(r\right)}$$

with

2b $$\rho_{k}\left(r,R_{k}\right)=\{\begin{array}{c} \frac{N_{el,k}}{\sigma_{k}\sqrt{2\pi }}\;\exp\left\{\frac{{-\left(r-R_{k}\right)}^{2}}{2\sigma_{k}^{2}}\right\}\;\mathrm{if}\;\left|r-R_{k}\right|\leq R_{c}\\ 0\;\mathrm{if}\;\left|r-R_{k}\right|>R_{c} \end{array}$$

where ρ_*j*_(**r**) is the Gaussian charge density centered on atom *j* in constraint region *c*, ρ_*k*_(**r**,**R**_*k*_) is the Gaussian charge density of atom *k*, *N*_*el*,*k*_ is the number of valence electrons on atom *k*, σ_*k*_ is the width parameter, **R**_*k*_ is the nuclear position of atom *k*, and *R*_c_ is the real-space cutoff radius (the location where the Gaussian is truncated to 0).^44^ The value of σ_*k*_ sets the degree of charge localization on atom *k* and needs to be calibrated for proper CDFT constraints (Section S3). Lastly, the excited-state minimum in eq 1 is achieved by first minimizing the DFT energy with respect to ρ, followed by maximizing with respect to *V*_c_.

Figure 1a presents the two diabatic Marcus PESs when transferring 1.0 *e* across the 4Au/3TiO_2_ interface. As illustrated in Figure 1a, Δ*G* was obtained from the energy difference between the 4Au^+1.0*e*^/3TiO_2_^–1.0*e*^ initial charge-separated state at the initial (excited state) geometry and the 4Au^0.0*e*^/3TiO_2_^0.0*e*^ final charge-recombined state at the final (ground state) geometry. And λ was obtained from the CDFT energy difference between the initial and final geometries, with both calculations having constraints in the initial charge-separated state (+1.0 *e* on 4Au and −1.0 *e* on 3TiO_2_).

**Figure 1:**
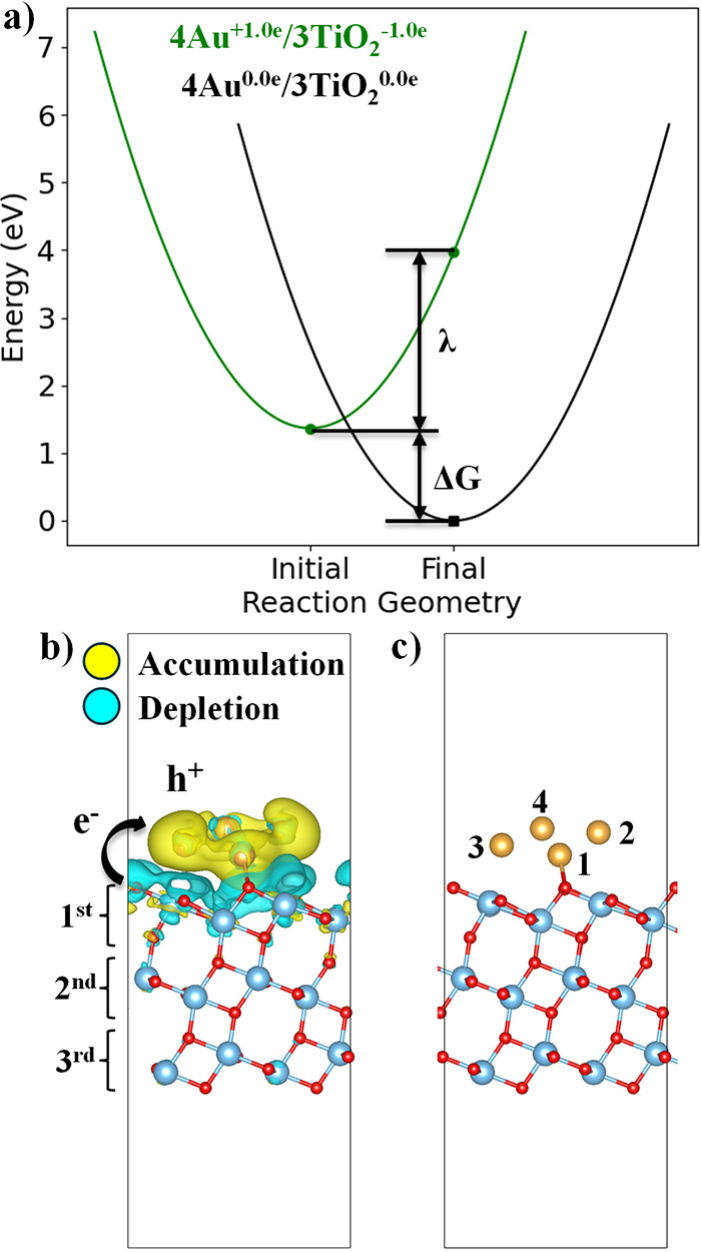
Marcus theory potential energy surfaces (PESs), charge density difference plot, and geometry of the 4Au/3TiO_2_ heterojunction. (a) Diabatic PESs of the 4Au^+1.0*e*^/3TiO_2_^–1.0*e*^ initial charge-separated state and 4Au^0.0*e*^/3TiO_2_^0.0*e*^ final charge-recombined state (or ground state). (b) Charge density difference map, final charge-recombined state minus initial charge-separated state, with an isovalue of 0.001 *e*/Å^3^ indicating charge recombination at the final geometry. (c) Structure of the final state geometry with Au atom indexing for Table 1.

Transferring 1.0*e* across the 4Au/3TiO_2_ interface results in the Marcus normal region with a Δ*G* and λ of −1.365 and 2.605 eV, respectively, leading to an activation energy barrier ( ${\Delta G}^{‡}=\frac{{\left(\lambda +\Delta G\right)}^{2}}{4\lambda }$ ) of 0.147 eV.

We can then leverage pre-established DFT analysis tools like charge density difference (CDD) plots and charge analysis schemes to examine charge recombination. Here, we focus our charge recombination analysis on the fixed, final geometry (Figure 1 and Table 1). As illustrated in Figure 1b, the CDD plot confirms charge density depletion from 3TiO_2_ and charge density accumulation on the 4Au cluster, consistent with expectations. Notably, we observe that charge density depletion is predominantly localized in the first layer of 3TiO_2_ and is quantified through the +0.956*e* difference between the final charge-recombined and initial charge-separated states (Table 1). Meanwhile, Hirshfeld charge analysis reveals that charge recombination occurs at the surface of the 4Au cluster. In particular, Au atoms 1, 2, and 3—those closest to 3TiO_2_ along the z direction (Figure 1c)—acquire the majority of the transferred charge. In contrast, Au atom 4—the furthest atom from 3TiO_2_—acquires the least amount of charge. After charge recombination, Au atoms 1 and 2 switch to negative values while Au atoms 3 and 4 remain positively charged, thus polarizing the 4Au cluster. An alternative analysis to complement Figure 1b and Table 1 is to also account for the effect of geometry change during charge recombination. Such results can be found in Figure S4 and Table S5 of Section S6. We find that including the effect of geometry change introduces additional complexity that obscures the direct comparison of the two charge states (Figure S4). However, our major findings remain consistent overall, except for the observation that the closest Au atom to 3TiO_2_ (Au atom 1) gains the largest quantity of charge (Table S5).

**Table 1:** Hirshfeld Charges of the Initial Charge-Separated and Final Charge-Recombined States and Their Differences (Final Minus Initial) at the Final Geometry

Hirshfeld (*e*)	4Au^+1.0*e*^/3TiO_2_^–1.0*e*^	4Au^0.0*e*^/3TiO_2_^0.0*e*^	Difference
Au atom 1	0.126	–0.134	–0.260
Au atom 2	0.271	–0.020	–0.291
Au atom 3	0.363	0.063	–0.301
Au atom 4	0.243	0.095	–0.148
4Au Cluster	1.003	0.004	–0.999
1st Layer of TiO_2_	–0.552	0.403	0.956
2nd Layer of TiO_2_	–0.429	–0.406	0.023
3rd Layer of TiO_2_	–0.018	0.002	0.020
3TiO_2_	–1.000	–0.001	0.999

Orbital charge density maps combined with atom-resolved projected density of states (PDOS) analyses offer insight into the dominant charge recombination pathway across the 4Au/3TiO_2_ interface. Here, we inspect the PDOS and the charge densities of the LUMO and HOMO of the initial charge-separated and final charge-recombined states, respectively, at the final geometry (Figure 2). Charge density is primarily localized on the 4Au cluster at the HOMO with minor charge density in the first layer of 3TiO_2_. For the LUMO, charge density is completely delocalized in 3TiO_2_. The overlap in charge densities in the first layer of 3TiO_2_ appears to facilitate charge recombination from the LUMO to the HOMO. Upon accounting for geometry changes, this charge density overlap persists in the first layer of 3TiO_2_ and additionally involves 4Au states (Figure S5).

**Figure 2:**
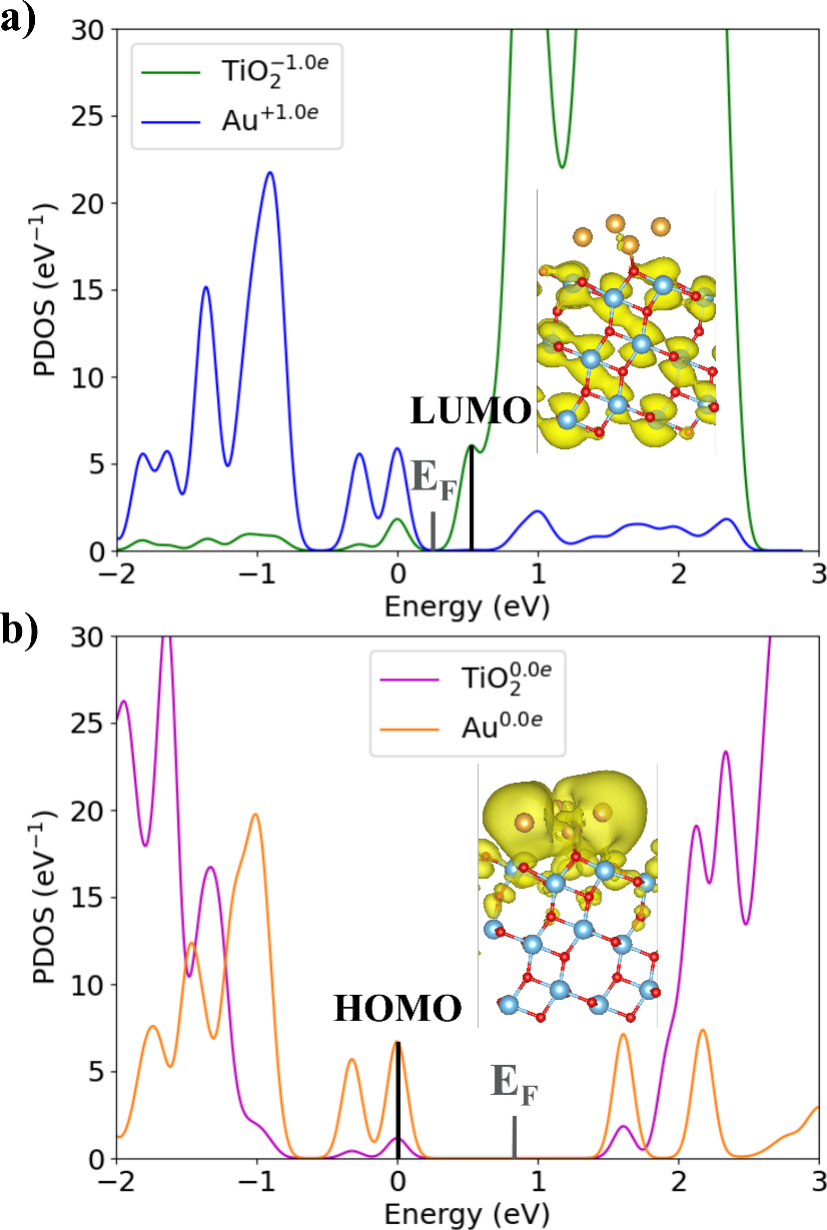
Atom-resolved PDOS before and after transferring 1.0*e* across the 4Au/3TiO_2_ interface aligned at the HOMO. Visualization of charge density in the LUMO and HOMO orbitals. (a) Atom PDOS and LUMO charge density plot of the Au^+1.0*e*^/3TiO_2_^–1.0*e*^ initial charge-separated state at the final geometry. (b) Atom PDOS and HOMO charge density plot of the Au^0.0*e*^/TiO_2_^0.0*e*^ final charge-recombined state at the final geometry.

According to the PDOS in Figure 2, charge recombination induces HOMO–LUMO gap renormalization from the Au^+1.0*e*^/3TiO_2_^–1.0*e*^ initial charge-separated state to the Au^0.0*e*^/TiO_2_^0.0*e*^ final charge-recombined state (Table 2). Since the atomic positions are fixed at the final geometry, this result suggests a strong exciton binding energy (*E*_b_) of 1.095 eV, which we determine from the difference in HOMO–LUMO gap between the initial and final states. However, when incorporating the PDOS from the initial geometry (Figure S5), the observed HOMO–LUMO gap renormalization is more likely attributed to bipolaron formation, driven by the relaxation of both the first TiO_2_ layer (−1.0*e*) and the 4Au atoms (+1.0*e*). Here, the bipolaron *E*_b_ is 0.277 eV (Table S6), indicating strong electron–hole attraction. The band structure renormalization observed in Figures 2a and S5a is consistent with the presence of large and medium-sized polarons in anatase TiO_2_.^45,46^ Moreover, the *V*_c_ and dipole moment (p) both decrease sufficiently to invert their directions when transitioning from the initial charge-separated state to final charge-recombined state, independent of geometry factors (Tables 2 and S6).

**Table 2:** HOMO–LUMO gap, *V*_c_, and Dipole Moment (*p*) of the Initial Charge-Separated State and Final Charge-Recombined State and Their Differences (Final Minus Initial) at the Final geometry

energy (eV)	4Au^+1.0*e*^/3TiO_2_^–1.0*e*^	4Au^0.0*e*^/3TiO_2_^0.0*e*^	difference
HOMO–LUMO gap	0.515	1.610	1.095
*V*_c_ (4Au cluster)	3.236	–0.058	–3.294
*p* (*e·*•Å) (*z*-direction)	2.012	–0.172	–2.184

To achieve an accurate prediction of charge transfer time scales, the appropriate Marcus theory rate expression must be determined for the Au/TiO_2_ system. In Marcus theory, there is both a nonadiabatic rate expression

3a $$k_{ET}^{non}=\upsilon_{el}\;\exp\left[-\frac{{\left(\lambda +\Delta G\right)}^{2}}{4\lambda k_{B}T}\right]$$

with

3b $$\upsilon_{el}=\frac{{H_{ab}}^{2}}{\hbar }\sqrt{\frac{\pi }{\lambda k_{B}T}}$$

and an adiabatic (Marcus–Levich–Dogonadze theory) rate expression

4 $$k_{ET}^{ad}=\upsilon_{n}\;\exp\left[-\frac{{\left(\lambda +\Delta G\right)}^{2}}{4\lambda k_{B}T}\right]$$

where υ_*el*_ is the electronic frequency, λ is the reorganization energy, Δ*G* is the free energy difference, *k*_*B*_ is the Boltzmann constant, *T* is the temperature of the nuclei, ℏ is the reduced Planck’s constant, and υ_*n*_ is the nuclear frequency.^47^ As prescribed by Landau–Zener theory, we identify the appropriate Marcus rate expression for our system through the electron transmission coefficient (κ_*el*_)­

5 $$\kappa_{el}=\frac{1-\exp\left[-\frac{\upsilon_{el}}{2\upsilon_{n}}\right]}{1-\frac{1}{2}\exp\left[-\frac{\upsilon_{el}}{2\upsilon_{n}}\right]}$$

where κ_*el*_ represents the probability an electron will either hop (κ_*el*_ ∼ 1) or tunnel (κ_*el*_ ∼ 0) from one state to another through the competition between υ_*el*_ and υ_*n*_.^47^ To use eq 5, *H*_*ab*_ is determined at a specific geometry through the mixed coupling method,

6 $$\left|H_{ab}\right|=\sqrt{\left(E-E_{A}\right)\left(E-E_{B}\right)}$$

where *E* is the adiabatic (or ground-state) DFT energy, *E*_A_ is the initial charge-separated state energy, and *E*_B_ is the final charge-recombined state energy.^31,48^ Generally, when *H*_ab_ is < *k*_B_ *T*, κ_*el*_ approaches zero and follows a nonadiabatic charge transfer process dictated by the magnitude of *H*_ab_ and Δ*G* ^‡^, following eq 3. In the adiabatic regime, κ_*el*_ approaches unity with an *H*_ab_ ≫ *k*_B_ *T*, and the charge transfer time scale is governed by υ_*n*_ and Δ*G* ^‡^ following eq 4.

For the 4Au/3TiO_2_ system, eq 6 predicts *H*_ab_ = 3.3*k*_B_ *T* at the final geometry, whereas *H*_ab_ computed from the original CDFT^31^ and Migliore wave function overlap^49^ methods are even larger (Table S4). Large *H*_ab_ persists across methods and geometries examined (Section S4). Consistent with these values, Figure 2 shows pronounced spatial overlap between the LUMO of the initial charge-separated state and HOMO of the final charge-recombined state in the first TiO_2_ layer. The system exhibits κ_*el*_ = 0.92, indicating sufficient thermal energy is available for electron hopping through eq 4.

For eq 4, the governing charge recombination υ_*n*_ is identified via complementary phonon mode calculation, which was found to be consistent with phonon influence spectrum analyses discussed later in the NAMD section. Phonon mode analysis using GPAW, summarized in Section S7 with Figures S6–S7 and Table S7, indicates that the υ_*n*_ most likely governing charge recombination across the 4Au/3TiO_2_ interface is 628 cm^–1^ (18.827 THz). We assign this mode as υ_*n*_ due to the O atom vibrational vectors pointing toward the Au cluster, most notably the O atom bonded to Au (Figure S7), consistent with our reaction coordinate in Section S5. Plugging this value into eq 4, we arrive at Table 3. Table 3 summarizes the charge recombination energetics, characteristic frequencies, and time scales obtained from CDFT + Marcus theory for the 4Au/3TiO_2_ system. The nonadiabatic recombination time scale (1/*k*_*ET*_^non^) is ≈4 ps, whereas the adiabatic value (1/*k*_*ET*_^ad^) is ≈16 ps. Because the two Marcus rate expressions share the same exponential factor, the difference arises solely from their prefactors, which are υ_*el*_ in the nonadiabatic case and υ_*n*_ in the adiabatic limit. Thus, accounting for adiabaticity lengthens charge recombination across the 4Au/3TiO_2_ interface by roughly a factor of 4 relative to the nonadiabatic scenario. Comparable time scale ratios, (1/*k*_*ET*_^ad^)/(1/*k*_*ET*_^non^) ≈ 2–40, have been reported for mixed-valence systems^50^ operating near the adiabatic regime (κ_*el*_ ≈ 1), supporting the plausibility of the 4:1 ratio observed here.

**Table 3:** CDFT + Marcus Theory Charge Recombination Energetics, Frequencies, and Time Scales of Transferring 1.0*e* across the 4Au/3TiO_2_ Interface

energetics (eV)				frequencies (THz)		time scales (ps)	
Δ*G*	λ	*H*_ab_	Δ*G* ^‡^	υ_*el*_	υ_*n*_	1/*k*_*ET*_^non^	1/*k*_*ET*_^ad^
–1.365	2.605	0.082	0.147	70.576	18.827	4.255	15.952

Next, we compare DFT results obtained with GPAW to AIMD simulations performed with VASP. These AIMD trajectories serve as inputs for the NAMD simulations. Figure 3a illustrates the dynamic behavior of the HOMO–LUMO gap in 4Au/3TiO_2_, ranging from 1.12 to 2.11 eV over a 6 ps AIMD trajectory at 300 K. The mean and standard deviation of the gap are 1.65 and 0.17 eV, respectively, consistent with the 0 K DFT calculated HOMO–LUMO gap at the final charge-recombined state (Table 2). In addition, the AIMD trajectory provides a phonon influence spectrum that quantifies the coupling strength of each vibrational mode to the electronic states (Figure 3b). These couplings set the relaxation and decoherence time scales observed in NAMD.^18^

**Figure 3:**
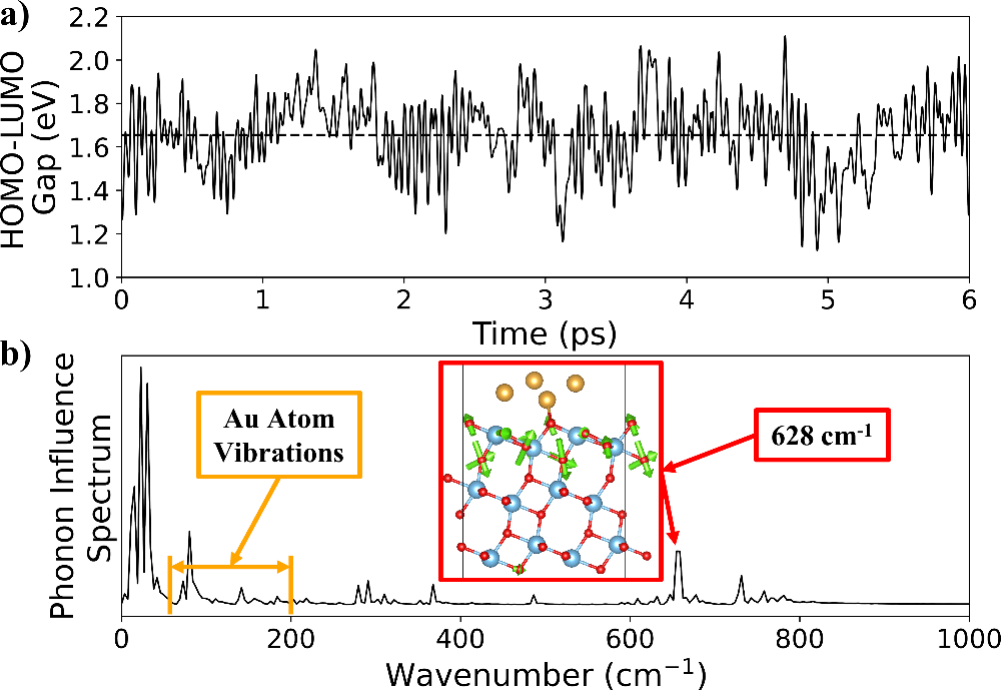
Comparison of VASP AIMD with GPAW DFT for the 4Au/3TiO_2_ system. (a) The HOMO–LUMO gap with respect to simulation time and (b) the phonon influence spectrum at 300 K using VASP. The dominant 628 cm^–1^ vibrational mode vector plot and orange bars indicating the window of Au atom vibrations are from the 0 K DFT frequency calculation using GPAW.

Figure 3b shows that the phonon mode analysis is consistent with the HOMO–LUMO phonon influence spectrum. In particular, our 0 K DFT frequency calculation of 4Au/3TiO_2_ with GPAW, documented in Figures S6 and S7, reveals that the Au atoms vibrate between 50 and 200 cm^–1^. This result explains the high intensity peaks in that frequency range in Figure 3b. Peaks above 200 cm^–1^ are attributed to TiO_2_ vibrations as indicated by the phonon mode analysis. Finally, the most prominent peak at 650 cm^–1^ lies near the selected υ_*n*_, and both are evident in Figure 3b.

In this section, we validate the results obtained from CDFT+Marcus theory with NAMD, which is a method that has successfully been applied to investigate electron–hole recombination in the 20Au/TiO_2_ system.^18^ In the NAMD simulations, we evaluated ground-state (Φ_0_) and excited-state (Φ_1_) electron populations in 4Au/3TiO_2_ using both the FSSH and DISH methods, respectively. Φ_1_ is defined as a two-state system where an excited electron is on the LUMO and the hole is on the HOMO. As illustrated by the charge density plots in Figure 4a, the HOMO localizes primarily on the 4Au cluster with minor contributions in the first layer of 3TiO_2_, whereas the LUMO is delocalized across the bottom two layers of 3TiO_2_. These VASP-derived HOMO and LUMO charge density plots in Figure 4a resemble those obtained using GPAW CDFT in Figure 2.

**Figure 4:**
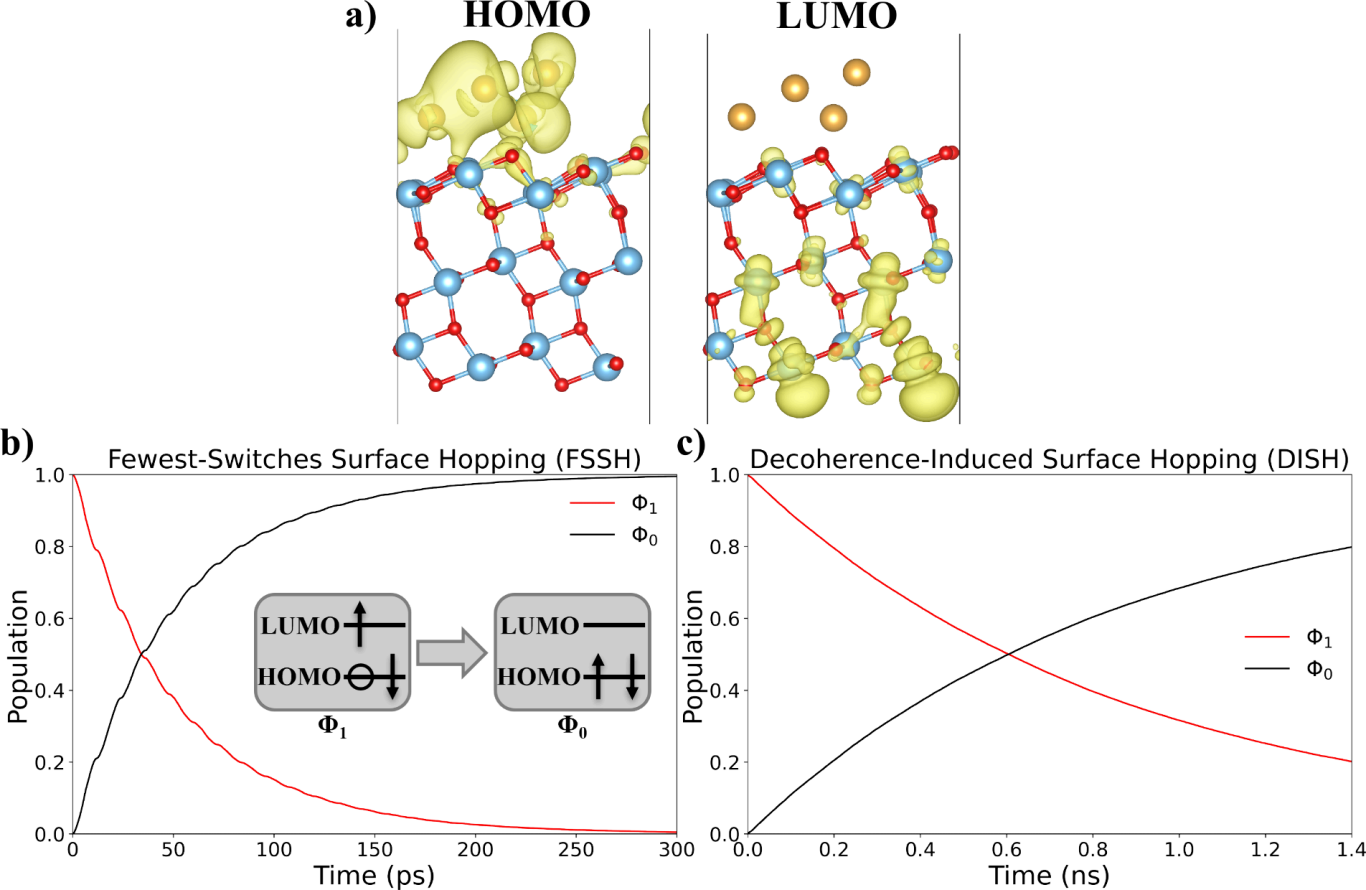
NAMD charge density maps, ground-state (Φ_0_) electron populations, and excited-state (Φ_1_) electron populations in the 4Au/3TiO_2_ system. (a) HOMO and LUMO charge density of a representative 4Au/3TiO_2_ structure using VASP at 300 K, where all results resemble what we obtained using GPAW (Figure 2). Both charge density plots use an isovalu*e* of 0.001 *e*/Å^3^. Electron population decay plots using (b) FSSH and (c) DISH.

Figure 4b and c demonstrate how the Φ_1_ electron population decays over time using the FSSH and DISH surface hopping techniques, respectively. With FSSH, the electron–hole recombination lifetime is 51 ps, as determined by fitting the energy decay with an exponential decay function (Figure S8 in Section S8). In comparison, nonadiabatic Marcus theory provides a 1/*k*_*ET*_^non^ of 4 ps (Table 3), which is about an order of magnitude shorter than the FSSH result. Additionally, we calculate a 4 fs dephasing time (Figure S9), meaning the characteristic time during which Φ_1_ loses quantum coherence is much faster than the predicted FSSH lifetime. The difference in lifetimes underscores the need for DISH to accurately predict electron–hole recombination time scales in 4Au/3TiO_2_. This observation is also consistent with our previous finding that the adiabatic eq 4 appropriately describes charge recombination in this system. DISH yields an electron–hole recombination lifetime of 860 ps (Figure S8), whereas adiabatic Marcus theory provides a 1/*k*_*ET*_^ad^ of 16 ps (Table 3). Although these numbers differ by roughly 2 orders of magnitude, such a gap is reasonable because the Marcus rate is exponentially sensitive to its driving parameters. In addition, DISH resolves orbital-specific dynamics, while CDFT is site/element-specific, so each method samples different aspects of the recombination process. Considering that adequate agreement between 1/*k*_*ET*_^ad^ and the DISH lifetime, and likewise between 1/*k*_*ET*_^non^ and the FSSH lifetime provides validation of our CDFT+Marcus theory approach.

Within the NAMD method, the DISH recombination lifetime is just above an order of magnitude longer than the FSSH lifetime. A similar trend emerges when using CDFT+Marcus theory where 1/*k*_*ET*_^ad^ is just under an order of magnitude longer than 1/*k*_*ET*_^non^ (Table 3). Notably, CDFT+Marcus theory recovers the NAMD recombination lifetime trends in the 4Au/3TiO_2_ system while demanding roughly 80% fewer node-hours and requires much less data storage than the NAMD simulations. In addition, the 16 ps time scale for 1/*k*_*ET*_^ad^ obtained from site-specific CDFT+Marcus theory is comparable to the previously reported recombination time scale of 60 ps from site-resolved trXPS measurements.^21^ However, this apparent quantitative agreement should be interpreted with caution, as the trXPS experiments were performed on substantially larger Au/TiO_2_ interfaces, containing on the order of 2 × 10^5^ Au atoms per NP.^21^ Therefore, the present CDFT+Marcus theory approach is sufficient to predict charge transfer trends across the Au/TiO_2_ heterojunction and at a fraction of the computational cost when compared to NAMD.

Since CDFT coupled with Marcus theory appropriately predicts charge transfer trends in Au/TiO_2_, we study how the size of the Au cluster influences charge recombination time scales. According to Marcus theory in a dielectric continuum, Δ*G* and λ generally scale as 1/*r*, where r is the radius of the charged region.^51,52^ In the Au/TiO_2_ system, for instance, transferring charge from 3TiO_2_ to a small Au cluster results in a large Δ*G* ^‡^, positioning the reaction into the Marcus normal region (Figure 5 and Table 4). As the Au cluster size increases from 4Au to 6Au, both Δ*G* and λ decrease by ∼36%. These reductions in Δ*G* and λ lower Δ*G* ^‡^, thereby shifting the reaction toward the Marcus top region. Coupled with a strong *H*_ab_, we hypothesize that there may be an optimal Au cluster size with a recombination time scale near the lower limit of 1/υ_*n*_ (Δ*G* ^‡^ ≈ 0). However, current CDFT calculations are unable to reach this limit under practical computational wall time.

**Figure 5:**
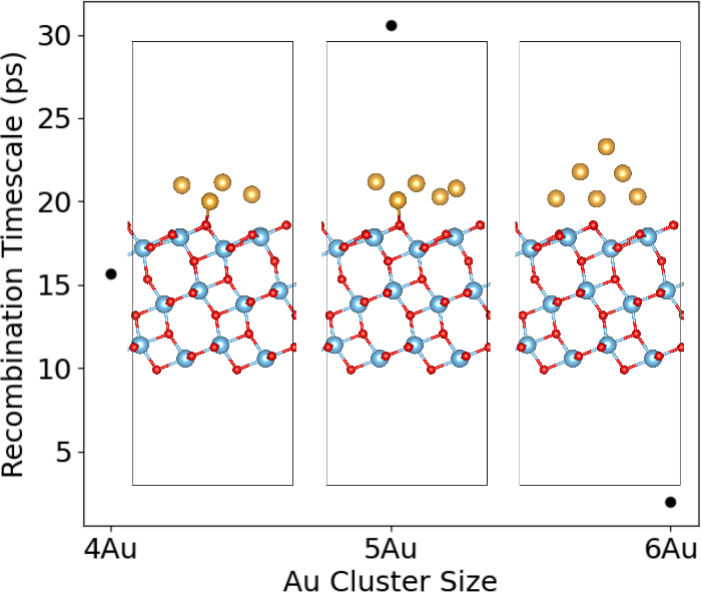
CDFT + Marcus theory adiabatic charge recombination time scales and Au^0.0*e*^/TiO_2_^0.0*e*^ final geometries when transferring 1.0*e* with respect to Au cluster sizes.

**Table 4:** CDFT + Marcus Theory Charge Recombination Energetics and Time Scales, the HOMO–LUMO Gaps of the Initial Charge-Separated and Final Charge-Recombined States at Their Respective Geometries, and the Bipolaron *E*_b_ for the 4Au, 5Au, and 6Au Clusters on 3TiO_2_

energy (eV)	4Au/3TiO_2_	5Au/3TiO_2_	6Au/3TiO_2_
Δ*G*	–1.365	–0.990	–0.877
λ	2.605	2.190	1.666
Δ*G* ^‡^	0.147	0.164	0.093
Initial state HOMO–LUMO gap	1.333	0.665	0.694
Final state HOMO–LUMO gap	1.610	0.691	1.625
Bipolaron *E*_b_	0.277	0.026	0.931
1/*k*_*ET*_^*ad*^	15.952	30.581	1.970

Notably, the 5Au cluster has an odd electron count (open-shell), leading to a partially occupied HOMO and reduced HOMO–LUMO gap (Table 4 and Figure S10). In this particular case, Δ*G* and λ fall by ∼28% and ∼16%, respectively, relative to the 4Au cluster. This energy shift raises Δ*G* ^‡^ and thus prolongs the charge recombination time scale. For the 5Au and 6Au clusters, λ scales roughly with the fraction of Au atoms relative to the 4Au cluster ( $\lambda =\frac{4\lambda_{4Au}}{N}$ ), approaching values of 4/5 and 2/3, respectively. These factors, however, are only observed when comparing across closed-shell systems for Δ*G* ( $\Delta G=\frac{4{\Delta G}_{4Au}}{N}$ , *N* even). In the 5Au cluster, the pronounced drop in Δ*G* is plausibly correlated with the substantial narrowing of the HOMO–LUMO gap, since orbital energy gaps are commonly used as first approximations for Δ*G*.^53^ Interestingly, λ is a geometric parameter and thus its trend remains invariant upon introducing open-shell character, whereas Δ*G*, containing both geometric and electronic contributions, exhibits a perturbed shift in an open-shell system. Additionally, the trend in bipolaron *E*_b_ is inversely proportional to the charge recombination time scale trend for the Au clusters.

Lastly, despite the promises of applying CDFT+Marcus theory to study complex interfacial charge transfer processes and the consistent trends with FSSH/DISH time scales, we note the following limitations of our current study. These limitations could arise from both CDFT and Marcus theory. For CDFT, SIE can bias *H*_ab_, where mitigation typically involves carefully defined CDFT constraints and the adoption of hybrid functionals.^31^ Here we calibrate the CDFT constraints (Section S3) and apply a Hubbard U correction (Section S4), which partially reduces *H*_ab_, but our current computational resources forbid us from adopting a full hybrid-functional calculation. In addition, the vibrational modes entering eq 4 were obtained from finite-displacement calculations using GPAW, which only capture phonons whose wavelengths are comparable to or smaller than the supercell.^54^ Long-wavelength phonons can be treated using reciprocal-space density functional perturbation theory (DFPT).^54,55^ To our knowledge, no currently available code combines DFPT with CDFT, but the potential-based CDFT framework^56^ is a natural route for such an extension. For Marcus theory, the assumption of harmonic PESs is most reliable for simple molecules.^28^ A one-dimensional reaction coordinate model (Section S5) indicates that the final charge-recombined PES is anharmonic, whereas the initial charge-separated PES is harmonic (Figure S4). Accordingly, absolute charge recombination time scales should be interpreted with caution; mechanistic assignments and relative trends are more robust within this framework.

In summary, we outlined a clear procedure of applying CDFT coupled with Marcus theory to predict charge recombination across the Au/TiO_2_ metal-SC interface and validated those results with well-established NAMD methods. Specifically, we modeled recombination from a charge-separated state to ground state in 4Au/3TiO_2_ with CDFT to obtain the Δ*G*, λ, and *H*_ab_ parameters for Marcus theory. We highlighted the advantages of applying these methods: (i) they facilitate assigning charges to specific fragments of atoms, (ii) they allow the investigation of the recombination pathway through established DFT analysis tools (e.g., Hirshfeld charge, Bader charge, charge density, and PDOS), and (iii) they use a simple physical framework, Marcus theory, to describe the recombination process. We identify a bipolaron in the charge-separated state, with recombination occurring primarily from the LUMO on 3TiO_2_ to the HOMO on 4Au through the charge density overlap in the first layer of 3TiO_2_. In addition, the Au atoms closest to the 3TiO_2_ surface gain the largest quantity of charge. The pronounced spatial overlap in charge density leads to a large *H*_ab_, driving the charge recombination process along the adiabatic Marcus theory pathway, which falls into the Marcus normal region. The time scales obtained from nonadiabatic and adiabatic Marcus rate expressions compare reasonably well with those obtained from the FSSH and DISH methods, respectively. Lastly, we explored how charge recombination time scales depend on Au cluster size. Our analysis shows that Δ*G* and λ scale as 4/n in closed-shell systems, yet it also highlights the challenge of forecasting Δ*G* in open-shell clusters, which is a difficulty that likely stems from their narrower HOMO–LUMO gaps. Notably, our explorations are constrained to a small window of Au cluster sizes due to the ∼100 atom limit in CDFT under practical computational wall time. However, we propose that our approach of applying CDFT + Marcus theory to the Au/TiO_2_ interface lays the groundwork for future site-specific charge transfer studies across other complex condensed matter interfaces.

Moving forward, the advent of exascale computing will enable real-space CDFT simulations of increasingly larger systems, potentially bridging the gap in charge transfer time scales between Marcus theory and experiments. Future computational advancements may enable a more complete understanding of the size-dependent nature of the Au cluster as well as the TiO_2_ SC. Additionally, a comprehensive mechanistic understanding of solvent environments, defect states, TiO_2_ phases and surfaces, and core–shell NP interactions in Au/TiO_2_ are currently lacking. We are optimistic that future studies will further elaborate on how to efficiently harvest charges by extending charge recombination time scales. These advancements will be critical for the effective deployment of Au/TiO_2_ in photocatalytic and photovoltaic applications.

## Supplementary Material







## Notes

Theoretical background of CDFT and NAMD; computational details of Au/TiO_2_ geometric optimization, Hubbard U correction, calibration of Hirshfeld width parameter; prediction of electronic coupling (Hab); verification and challenges of Marcus theory; analyses of charge recombination at the initial and final geometries; phonon modes for adiabatic charge recombination; details of electron–hole recombination with NAMD; PDOS visualization of the 5Au/3TiO_2_ system (PDF)
All GPAW CDFT calculation inputs and outputs (ZIP)
Transparent Peer Review report available (PDF)
