# Supplementary material for: Real-Space Constrained Density Functional Theory Investigation of Site-Specific, Interfacial Charge Recombination Dynamics Across the Au Nanoparticle/TiO2 Heterojunction
Source: J Phys Chem Lett. 2026 Jan 6;17(2):570–8. doi: 10.1021/acs.jpclett.5c02905 (PMC12814526; doi:10.1021/acs.jpclett.5c02905)
Supplement: Supplementary file 1 [file jz5c02905_si_001.pdf]

Supplementary Information: Real-Space Constrained DFT Investigation of Site-Specific, Interfacial Charge Recombination Dynamics Across the Au Nanoparticle/TiO_2 Heterojunction

Drew M. Glenna#[a, b], Carlos Mora Perez[b], Ernest Hermosillo[b], Haiyan Zhao*[a, c], and Jin Qian*[b]

[a] Department of Nuclear Engineering & Industrial Management, Center for Advanced Energy Studies, University of Idaho, Idaho Falls, ID 83402, USA
[b] Chemical Sciences Division, Lawrence Berkeley National Laboratory, Berkeley, CA 94720, USA
[c] Department of Chemical and Biological Engineering, Center for Advanced Energy Studies, University of Idaho, Idaho Falls, ID 83402, USA
E-mail: haiyanz@uidaho.edu and jqian2@lbl.gov

Table of Contents

S1: Theoretical Methods 2

S1.1: Au/TiO_2 Geometry 3

S1.2: CDFT Methods 3

S1.3: Obtaining ΔG, λ, and Hab with CDFT 3

S1.4: VASP and NAMD Methods 4

S2: Hubbard U Correction in Bulk Anatase TiO_2 6

Figure S1. Bandgap energies with respect to Hubbard U correction values in bulk anatase TiO_2 7

S3: Calibration of Hirshfeld Width Parameters for CDFT Constraints 7

Table S1. Matching Bader and Hirshfeld charges to determine σAu in the 4Au/3TiO_2 system 7

Table S2. Matching Bader and Hirshfeld charges to determine σTi in the 4Au/3TiO_2 system 8

Table S3. Matching Bader and Hirshfeld charges to determine σO in the 4Au/3TiO_2 system 8

S4: Predicting Electronic Coupling (Hab) in 4Au/3TiO_2 9

Table S4. Methods for predicting Hab for 1.0 *e* charge recombination across the 4Au/3TiO_2 interface at the PBE+U+D3 level and at the final geometry 9

Figure S2. Hab energies with respect to Hubbard U correction values in the 4Au/3TiO_2 transition state geometry at the PBE+U+D3 level 11

S5: Verification and Challenges of Marcus Theory for Au/TiO_2 11

Figure S3. Diabatic potential energy surfaces (PESs) with respect to the vertical Au/TiO_2 separation ($\Delta z = z4Au - z1st - \text{layer TiO2}$) 12

S6: Analyses of Charge Recombination at the Initial and Final Geometries 13

Figure S4. Charge density difference map and geometries of $4Au/3TiO_2$ 13

Table S5. Hirshfeld charges of the initial charge-separated and final charge-recombined states and their differences (final minus initial) at their respective geometries 14

Figure S5. Visualization of charge density in the LUMO and HOMO and atom PDOS (aligned at the HOMO) in the $4Au/3TiO_2$ system 15

Table S6. HOMO−LUMO gap, V_C, and dipole moment (p) of the initial charge-separated state at the initial geometry and final charge-recombined state at the final geometry and their differences (final minus initial) 15

S7: Determining υ_n for Adiabatic Charge Recombination 16

Figure S6. Au atom phonon modes (υ) in the $4Au/3TiO_2$ system at the final charge-recombined state and final geometry 16

Figure S7. Potential charge recombination phonon modes (υ) in the $4Au/3TiO_2$ system at the final charge-recombined state and final geometry 17

Table S7. Adiabatic charge recombination timescales across the $4Au/3TiO_2$ interface with respect to phonon modes (υ) and their corresponding υ 17

S8: Electron-Hole Recombination with NAMD 17

Figure S8. Energy decay of the excited state ($\Phi 1$) electron population in the $4Au/3TiO_2$ system with FSSH (left) and DISH (right) at 300 K 18

Figure S9. The dephasing function (top) and autocorrelation function (bottom) with respect to time in the VASP AIMD $4Au/3TiO_2$ systems at 300 K 18

S9: Atom PDOS of the $5Au/3TiO_2$ System 18

Figure S10. Visualization of the atom PDOS in the $5Au/3TiO_2$ system aligned at the HOMO-1 19

S10: GPAW Structures, Inputs, and Outputs 19

S1: Theoretical Methods

The Vienna Ab initio Simulation Package (VASP[1-4]) is used to conduct ab initio molecular dynamics (AIMD) simulations and to determine the lattice dimensions for the $4Au/3TiO_2$ system. The lattice dimensions are obtained from a bulk TiO_2 volume relaxation density functional theory (DFT) calculation. The Grid-Based Projector-Augmented Waves (GPAW[5-8]) software is employed to conduct real-space DFT and constrained DFT (CDFT[9]) calculations to obtain the charge-recombined ground-state and the charge-separated excited-state energetics, respectively. Marcus theory[10] is used to predict the charge recombination timescales, which are compared with the recombination lifetimes from the more computationally demanding nonadiabatic molecular dynamics (NAMD) simulations. The following subsections provide details on the Au/TiO_2 cell geometry, the DFT and CDFT calculations, the procedure for predicting charge recombination timescales, and the NAMD approaches.

S1.1: Au/TiO_2 Geometry

The Atomic Simulation Environment (ASE[11]) software is used to construct all simulation models. First, we optimize the bulk anatase TiO_2 lattice parameters of the primitive cell within VASP, at the PBE+D3 level. We determined the a/b direction to be 3.81 Å and the c direction to be 9.77 Å, which agrees with the experimental lattice constants a/b = 3.78 Å and c = 9.51 Å at 300 K[12]. This bulk structure is used to construct a 3-layer anatase TiO_2 (101) surface slab, with a 15 Å vacuum gap along the z-axis between slabs, which is expanded to a 1×2×1 supercell to accommodate the Au atom clusters and minimize Au-Au periodic image interactions. In all cases, the lowest two layers of TiO_2 are kept frozen in their bulk positions. The 4 Au atom cluster was placed on top of a 3-layer anatase TiO_2 (101) slab (4Au/3TiO_2). The 4Au/3TiO_2 cell dimensions are 10.46 × 7.58 × 28.83 $Å^3$, which was used in the DFT and CDFT calculations in GPAW as well as in the NAMD simulations.

S1.2: CDFT Methods

All of the DFT and CDFT calculations carried out in GPAW use real-space finite-difference (FD) grids with a 0.20 Å grid spacing, a dipole correction in the z direction, are spin-polarized, at the Γ-point, incorporate a Fermi-Dirac smearing width of 0.10 eV, and employ three steps of Davidson diagonalization per self-consistent field (SCF) iteration. Long-range London dispersion interactions are accounted for with Van der Waals (vdW) forces by the Grimmes D3[13] corrections. Valence electron eigenfunctions are modeled by plane-wave basis sets with the projector-augmented (PAW[4,14]) method along with the Perdew-Burke-Enzerhof (PBE[15]) generalized gradient approximation exchange functional. An effective Hubbard U correction U_{eff} = 7.0 eV was applied to the Ti 3d orbitals to replicate the experimentally observed bulk anatase TiO_2 bandgap (see **Figure S1** in **Section S2**). For the SCF cycle, we employed GPAW's implementation of MixerDif, which is implemented with a 0.01 linear mixing coefficient, 1 old density, 100% density mixing weight, 0.01 linear magnetic mixing coefficient, 8 old magnetic densities, and 100% magnetic density mixing weight. The eigenstates, electron density, and total energy convergence thresholds for self-consistency are set to 1.0×10^{-10} eV^2/electron, 1.0×10^{-5} electrons/electron, and 1.0×10^{-6} eV/electron, respectively. The force convergence threshold for all DFT and CDFT ionic relaxation calculations are set to be 0.05 eV/Å. Periodic boundary conditions (PBC) are imposed in the x and y directions, whereas the z direction contains a non-PBC.

S1.3: Obtaining ΔG, λ, and H_{ab} with CDFT

First, ground-state DFT ionic relaxation calculations are performed at the PBE+D3 level in GPAW for the 4Au cluster and 3TiO_2 slab, separately. All Au atoms are allowed to move, whereas only the uppermost TiO_2 layer atoms are relaxed. Once the geometries of the 4Au cluster and 3TiO_2 are converged at the final charge-recombined state (or ground-state), the optimized 4Au cluster is placed on top of the optimized 3TiO_2 cell, 3 Å above the top layer of 3TiO_2. This 4Au/3TiO_2 structure is ionically relaxed to achieve the ground-state geometry, i.e., final geometry. Next, σ_k

is determined for each element by matching the total Bader[16-19] and Hirschfeld[20] charges in the 4Au cluster and $3TiO_2$ at the PBE+U+D3 level and final geometry (see **Tables S1-S3** in **Section S3**). This step provides the proper CDFT settings, σ_{Au}, σ_{Ti}, and σ_O of 0.61, 0.82, and 0.70 Å, respectively, to proceed with the initial charge-separated state calculations. It also supplies the final charge-recombined state CDFT and DFT (ground-state) energies at the final geometry. Similarly, the initial charge-separated state CDFT ionic relaxation calculations are performed at the PBE+D3 level for the isolated 4Au cluster and $3TiO_2$ slab, separately with charged cells and PBCs in all directions. Once the charge-separated state geometries of the isolated 4Au cluster and $3TiO_2$ slab are converged, the optimized 4Au cluster is placed on top of the $3TiO_2$ slab, 3 Å above the top layer of $3TiO_2$. This $4Au/3TiO_2$ structure is further relaxed to provide the initial charge-separated state geometry, i.e., initial geometry. Then, two single-point initial charge-separated state (+1.0 e on 4Au and −1.0 e on $3TiO_2$) CDFT calculations are conducted at both the initial and final geometries at the PBE+U+D3 level. The energy difference between the final charge-recombined state DFT energy at the final geometry and the initial charge-separated state CDFT energy at the initial geometry is ΔG. The energy difference between the initial and final geometries, with both containing the initial charge-separated state charge constraint is λ. The electronic coupling (H_{ab}) is determined by the mixed coupling method in the final geometry (see full discussion and **Table S4** in **Section S4**). Both the nonadiabatic and adiabatic Marcus theory rate expressions are used to estimate charge recombination timescales, as described in the main paper. Lastly, the verification and challenges of using Marcus theory to predict charge transfer timescales across the Au/TiO_2 interface are discussed in **Section S5**.

S1.4: VASP and NAMD Methods

VASP DFT and AIMD calculations were employed to benchmark the CDFT results. The PBE functional was used for all systems. The following PAW pseudopotentials were employed: PAW_PBE Ti 08Apr2002, PAW_PBE O 08Apr2002, and PAW_PBE Au 04Oct2007[4]. In all static calculations, the energy cutoff for the plane-wave basis set was converged to 600 eV (ENCUT = 600), which is 1.5 times larger than the largest cutoff value specified in the pseudopotential files, to ensure complete convergence of the total energy for the systems. The electronic SCF cycle was set to an energy convergence of 1.0×10^{-8} eV (EDIFF). During geometry optimization, the convergence criteria for all atomic forces are set to <0.01 eV/Å (EDIFFG = -0.01), and the Brillouin zone (BZ) is sampled using a Γ-point k-grid. Similar to our GPAW setup, we applied a Hubbard U_{eff} = 6.9 eV to the Ti 3d states using a simplified DFT+U approach[21] for all VASP calculations, which was determined to best replicate the experimentally observed bulk TiO_2 bandgap[22,23]. All calculations include a dipole correction that is in the surface normal (c-direction) direction (IDIPOL=3).

The AIMD ground-state simulations were conducted using a 1×1×1 Γ-centered k-point mesh. The energy cutoff for the plane-wave basis set was converged to 600 eV (ENCUT = 600) and a time step of 1 fs (POTIM = 1) was used with the Nose-Hoover thermostat. The geometry-optimized structures served as the starting point for the MD simulations. Initially, the two systems are thermally equilibrated for 3 ps under the canonical ensemble (NVT), with the velocities scaled

every 10 fs to room temperature. This is followed by a 10 ps production run, where the microcanonical ensemble (NVE) generates the trajectories at 300 K. The last 6 ps segment of the trajectory is selected for further analysis with NAMD methods.

The NAMD simulations of electron-hole recombination in the Au/TiO_2 system utilize both the fewest-switches surface hopping (FSSH[24]) and decoherence-induced surface hopping (DISH[25]) approaches. For FSSH, the nonadiabatic coupling (NAC) governs the hopping probability between two many-body states Φ_i and Φ_j and is given by

$$P_{i \to j}(t; \mathrm{d}t) = \int_t^{t+dt} \frac{2}{\|c_i(t)\|^2} Re\left[(\frac{id_{ij}}{\hbar}) c_i^*(t) c_j(t) \right] \mathrm{d}t \qquad \text{(S1)}$$

where d_{ij} (or NAC) is the off-diagonal term (nonzero when Φ_i and Φ_j differ by one KS orbital and zero otherwise), $c_i(t)$ is the time-dependent coefficient of Φ_i, $c_j(t)$ is the time-dependent coefficient of Φ_j, and $\hbar$ is the reduced Planck's constant[24]. A negative probability is not allowed and is therefore reset to zero. The hopping probability is thus induced by the strength of the electron-phonon coupling. Decoherence is included if the decoherence time is faster than the time of quantum transitions. The decoherence times are evaluated as the pure-dephasing times of the optical response theory. The pure-dephasing function is obtained using the second-order cumulant approximation, given as

$$D_{ij}(t) = \left[-\frac{1}{\hbar^2} \int_0^t dt' \int_0^{t'} dt'' C_{ij}(t'') \right] \qquad \text{(S2)}$$

where, $C_{ij}(t)$ is the energy gap autocorrelation function given in **eq S6**[26]. This is the case for electron-hole recombination in Au/TiO_2, as the electron-hole recombination is expected on the picosecond (ps) timescale, and the decoherence time is on the femtosecond (fs) timescale[27]. The DISH method employed is

$$t_i(t) > \tau_i(t) \qquad \text{(S3a)}$$

where $t_i(t)$ is the physical time (zero at the start of the NAMD simulation) and $\tau_i(t)$ is the time since the last decoherence event of state i, defined as

$$\frac{1}{\tau_i}(t) = \sum_{j \neq i}^{N} |c_i(t)|^2 r_{ij} \qquad \text{(S3b)}$$

where r_{ij} is the decoherence rates of each pair of states and is computed prior to the NAMD runs using the optical response theory[25]. The nonadiabatic and adiabatic electron-hole recombination timescales computed with NAMD are with the FSSH and DISH methods, respectively. Derivations

of **eq's S1** and **S3** can be found in previous NAMD studies that have employed the FSSH[24] and DISH[25] methods, respectively. In both FSSH and DISH methods, the probability of the electron hopping upward in energy is scaled by the Boltzmann factor:

$$\exp\left(-\frac{E_i - E_j}{k_B T}\right), E_i - E_j > 0 \quad \text{(S4)}$$

while the probability of hopping downward in energy remains unchanged, a more convenient method than rescaling velocities at each hop. Both the FSSH and DISH calculations are performed using the LIBRA[28] package, which utilizes DFT calculations and MD trajectories from VASP. The phonon modes that couple the HOMO−LUMO states of the heterojunction are determined by taking the Fourier transform of the autocorrelation function, resulting in the phonon influence spectrum. The energy gap autocorrelation function is defined as

$$C_{ij}(t) = \frac{\langle(\Delta E_{ij}(\mathbf{R}(t)) - \langle\Delta E_{ij}(\mathbf{R}(t))\rangle_T)\cdot(\Delta E_{ij}(\mathbf{R}(t_0)) - \langle\Delta E_{ij}(\mathbf{R}(t_0))\rangle_T)\rangle_T}{\langle(\Delta E_{ij}(\mathbf{R}(t_0)) - \langle\Delta E_{ij}(\mathbf{R}(t_0))\rangle_T)^2\rangle_T} \quad \text{(S6)}$$

for the state energy $E_{ij}(\mathbf{R}(t))$, where the brackets $\langle\ldots\rangle$ indicate canonical averaging[25]. A rapid decay of $C(t)$ from 1 to 0 indicates that fluctuations are driven by many vibrations, while periodic oscillatory behavior indicates that few modes are coupled to the electronic subsystem.

S2: Hubbard U Correction in Bulk Anatase TiO_2

The effective Hubbard U correction (U_{eff}) for Ti 3d states was calibrated in bulk anatase TiO_2 and applied in all the DFT and CDFT calculations using GPAW in this work. A ground-state DFT calculation at the PBE level produces a bulk anatase TiO_2 bandgap of 2.0 eV. **Figure S1** shows that a U_{eff} = 7.0 eV results in a bandgap of 3.20 eV, which agrees with the reported experimental bandgap of anatase TiO_2[23]. Accordingly, all the DFT and CDFT calculations in GPAW at the PBE+U+D3 level implement this U_{eff} = 7.0 eV.

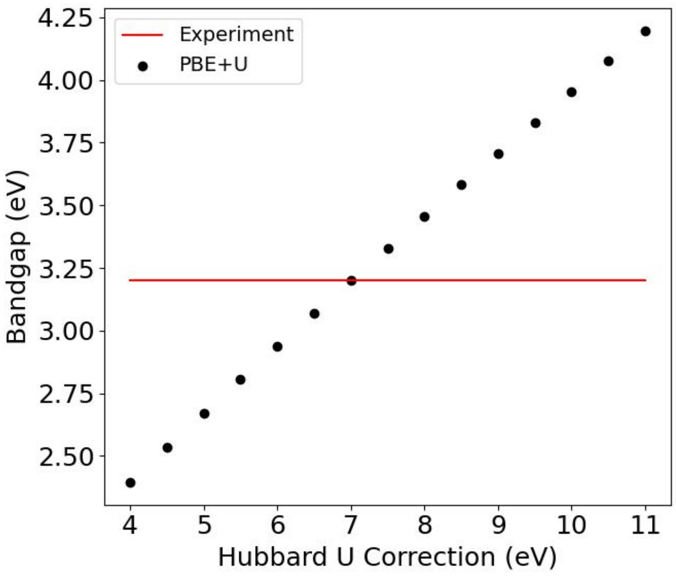

Figure S1. Bandgap energies with respect to Hubbard U correction values in bulk anatase TiO_2. The U_{eff} = 7.0 eV successfully reproduces the 3.20 eV experimental bandgap of bulk anatase TiO_2.

S3: Calibration of Hirshfeld Width Parameters for CDFT Constraints

A careful choice of CDFT constraint, i.e. underlying weight function scheme (w_c in **eq's 1** and **2**), is essential for obtaining reasonable energetics and forces, and thus physically meaningful charge state geometries[9]. In practice, real-space charge partitioning schemes like Becke and Hirshfeld perform the best[9]. Within GPAW, Hirshfeld w_c's are implemented; however, the effective constraint still depends on practical details of the reference charge densities, so tuning element-specific width parameters can be used to improve the fidelity of the targeted charge states. Here, we determined the Hirshfeld width parameter values of Au, Ti, and O (σ_{Au}, σ_{Ti}, and σ_O) by matching the Bader and Hirshfeld charges in the $4Au^{0.0e}/3TiO_2^{0.0e}$ final charge-recombined state at the final geometry. The CDFT calculation's charge constraint was set to $4Au^{0.0e}/3TiO_2^{0.0e}$ with a charge tolerance of 0.01 e, which is based on the Bader charge configuration in the ground-state DFT calculation at the PBE+U+D3 level. The total Hirshfeld and Bader charges on the 4Au (and $3TiO_2$) were converged with a σ_{Au} value of 0.61 Å, leading to a minimum total CDFT energy of −528.956 eV (see **Table S1**). This result is within 2 meV of the adiabatic (or ground-state) DFT energy (−528.958 eV) at the final geometry. Additionally, the default σ_{Ti} and σ_O values are reasonable settings for the $4Au/3TiO_2$ system (see **Tables S2** and **S3**). Thus, we use σ_{Au} = 0.61 Å, σ_{Ti} = 0.82 Å, and σ_O = 0.70 Å for all CDFT calculations in the main text. Lastly, we note that calibration of the Hirshfeld σ_ks yields more reliable charge state geometries and systematically lowers H_{ab} by bringing the charge state energies into closer agreement with the adiabatic (ground-state) curve (see **Section S4**).

Table S1. Matching Bader and Hirshfeld charges to determine σ_{Au} in the $4Au/3TiO_2$ system. The CDFT calculations are at the PBE+U+D3 level, in the $4Au^{0.0e}/3TiO_2^{0.0e}$ final charge-recombined state, and at the final geometry. The total Bader and Hirshfeld charges on the 4Au cluster are shown and bold indicates the most stable σ_{Au}.

σ_{Au} (Å)	Bader (e)	Hirshfeld (e)	Total CDFT Energy (eV)
0.68	315.161	316.000	−526.113
0.67	315.252	316.000	−526.882
0.66	315.390	316.000	−527.518
0.65	315.529	316.000	−528.066
0.64	315.651	316.000	−528.456
0.63	315.770	316.000	−528.733
0.62	315.888	316.000	−528.901
**0.61**	**316.001**	**316.000**	**−528.956**
0.60	316.122	316.000	−528.890

Table S2. Matching Bader and Hirshfeld charges to determine σ_{Ti} in the 4Au/3TiO_2 system. The CDFT calculations are at the PBE+U+D3 level, in the $4Au^{0.0e}/3TiO_2^{0.0e}$ final charge-recombined state, and at the final geometry. The total Bader and Hirshfeld charges on the 4Au cluster are shown and bold indicates the most stable σ_{Ti}.

σ_{Ti} (Å)	Bader (e)	Hirshfeld (e)	Total CDFT Energy (eV)
0.86	316.126	316.000	−528.883
0.84	316.067	316.000	−528.936
**0.82**	**316.001**	**316.000**	**−528.956**
0.80	315.957	316.000	−528.948
0.78	315.899	316.000	−528.913

Table S3. Matching Bader and Hirshfeld charges to determine σ_O in the 4Au/3TiO_2 system. The CDFT calculations are at the PBE+U+D3 level, in the $4Au^{0.0e}/3TiO_2^{0.0e}$ final charge-recombined state, and at the final geometry. The total Bader and Hirshfeld charges on the 4Au cluster are shown and bold indicates the most stable σ_O.

σ_O (Å)	Bader (e)	Hirshfeld (e)	Total CDFT Energy (eV)
0.74	316.086	316.000	−528.922
0.72	316.052	316.000	−528.944
**0.70**	**316.001**	**316.000**	**−528.956**
0.68	315.970	316.000	−528.952

0.66	315.933	316.000	−528.936

S4: Predicting Electronic Coupling (H_{ab}) in 4Au/3TiO$_2$

In Marcus theory, H_{ab} is the most difficult parameter to predict through CDFT methods. We tested the original CDFT[9], Migliore wave function overlap[29], and mixed coupling[9,30] methods to predict H_{ab} in the 4Au/3TiO$_2$ system. Each method is used at a specific geometry. For the equations in **Table S4**, E is the adiabatic (or ground-state) DFT energy, E_A is the 4Au$^{+1.0e}$/3TiO$_2^{-1.0e}$ initial charge-separated state energy, E_B is the 4Au$^{0.0e}$/3TiO$_2^{0.0e}$ final charge-recombined state energy, S_{AB} is the overlap integral between states A and B, ΔE_{AB} is the energy gap between the states ($\Delta E_{AB} = E_A - E_B$), Φ_A is the Kohn-Sham (KS) wave function of state A, Φ_B is the KS wave function of state B, V_A is the constraining potential of state A (see **eq 1**), V_B is the constraining potential of state B, w_A is the weight function of state A, w_B is the weight function of state B, and the overlap of each state with the adiabatic DFT state is $a \equiv \langle \Phi_A \mid \Phi_0 \rangle$ and $b \equiv \langle \Phi_B \mid \Phi_0 \rangle$[9]. All methods predict a large H_{ab} at the final (ground-state) geometry (see **Table S4**). In addition, large H_{ab} is predicted at both the initial (charge-separated) and transition state geometries (see **Sections S5** and **S6**). For the initial (charge-separated) geometry, the Migliore wave function overlap and mixed coupling methods result in 9.081 and 1.052 eV, respectively. Similarly for the transition state geometry, the Migliore wave function overlap and mixed coupling method values are 1.997 and 1.097 eV, respectively. Overall, we consistently predict large H_{ab} across methods and geometries in the 4Au/3TiO$_2$ system.

Table S4. Methods for predicting H_{ab} for 1.0 e charge recombination across the 4Au/3TiO$_2$ interface at the PBE+U+D3 level and at the final geometry. The preferred method is in bold.

Method	Equation	H_{ab} (eV)
Original CDFT	$H_{ab} = \frac{E_A + E_B}{2} S_{AB} - \left\langle \Phi_A \left\| \frac{V_A w_A + V_B w_B}{2} \right\| \Phi_B \right\rangle$	2867.746
Migliore	$H_{ab} = \left\| \frac{ab}{a^2 - b^2} \Delta E_{AB} \left(1 + \frac{a^2 + b^2}{2ab} S_{ab} \right) \frac{1}{1 - S_{AB}^2} \right\|$	6.541
**Mixed Coupling**	$\boldsymbol{\|H_{ab}\| = \sqrt{(E - E_A)(E - E_B)}}$	**0.082**

The original CDFT developers demonstrated that H_{ab} can be sensitive to the treatment of exchange and that hybrid functionals represent the gold standard for mitigating over delocalization[30]. In GPAW's FD mode, two available hybrid functionals are EXX (100% exact exchange) and PBE0 (25% exact exchange). For the present 4Au/3TiO$_2$ periodic model, both EXX and PBE0 CDFT calculations proved intractable. Ionic relaxation did not converge with the DFT total energy oscillating in the eV range after 48 hours on 2 nodes (512 cores), which is the limit of our computational wall time limit on NERSC. We note that per GPAW's documentation, these hybrid functionals remain untested for periodic DFT in FD mode, and current implementations lack the

parallel efficiency required for our system size. Given these limitations, we employed PBE+U+D3 as the most computationally feasible and stable option currently available for real-space CDFT calculations of this scale. While the influence of the Hubbard U correction on H_{ab} has not yet been systematically benchmarked against hybrid functionals, the +U term mitigates over delocalization in transition-metal d states, reproduces the experimental bandgap of bulk anatase TiO_2 (see **Figure S1** in **Section S2**), and has been reported to reduce H_{ab} in polaron hopping studies in both rutile and anatase TiO_2[31]. We further assessed the +U dependence of H_{ab} at the transition state geometry (see **Sections S5** and **S6**) of the 4Au/3TiO_2 system. As shown in **Figure S2**, increasing U_{eff} from 0.0 to 5.0 eV lowers H_{ab} by ~40.0 meV. Beyond U_{eff} = 5.0 eV the CDFT coupling is effectively converged, varying by only ~25.0 meV with U_{eff} = 5.0-30.0 eV. Accordingly, the choice U_{eff} = 7.0 eV determined in **Section S2** provides a reasonable compromise, simultaneously reproducing the bulk anatase TiO_2 bandgap and yielding a converged H_{ab}. Lastly, we note that U_{eff} > 7.0 eV produces unphysical density of states and is employed here solely to verify convergence of H_{ab}.

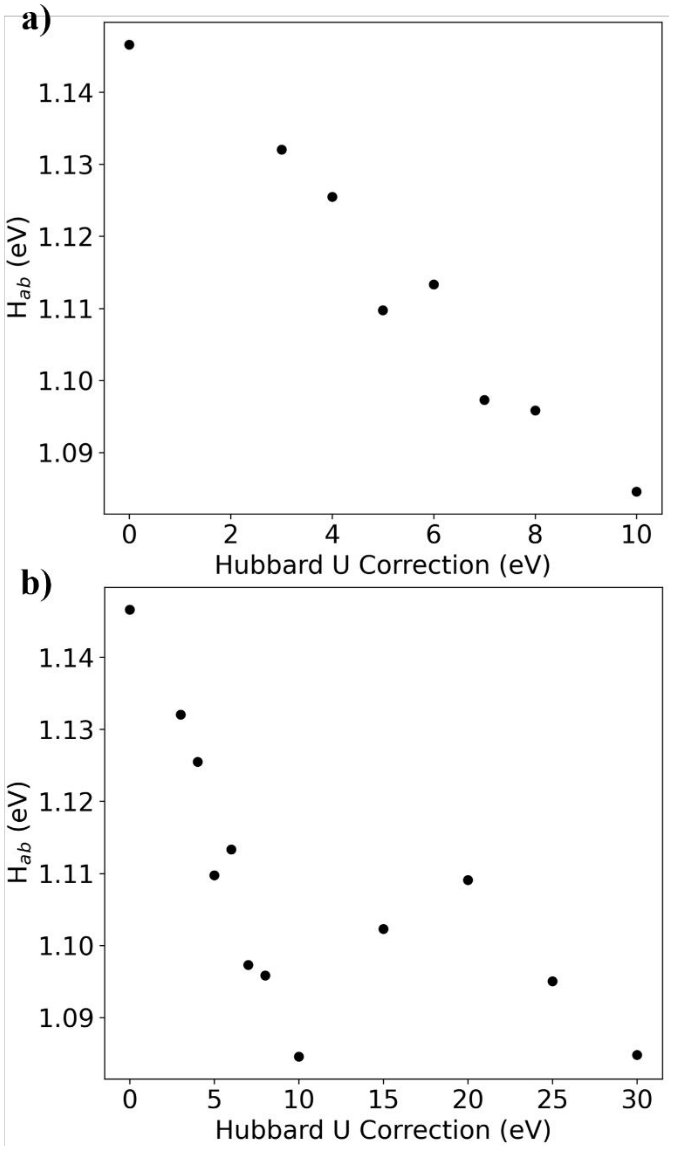

Figure S2. H_{ab} energies with respect to Hubbard U correction values in the 4Au/3TiO_2 transition state geometry at the PBE+U+D3 level. (a) H_{ab} energies with respect to physically reasonable Hubbard U correction values. (b) H_{ab} energies with respect to all tested Hubbard U correction values. The mixed coupling method in **Table S4** is used for computing H_{ab}.

S5: Verification and Challenges of Marcus Theory for Au/TiO_2

Marcus theory assumes harmonic potential energy surfaces (PESs), an approximation that is generally adequate for simple molecules[10]. In complex, heterogeneous systems, however, appreciable anharmonicities can arise. We therefore evaluated the harmonicity of the 4Au/3TiO_2 system by mapping the PES's of the initial charge-separated and final charge-recombined states along a simple linear reaction coordinate, following prior CDFT studies of long-range charge transfer[30,32]. The reaction coordinate is defined as the vertical Au/TiO_2 separation, $\Delta z = \langle z \rangle_{4Au} - \langle z \rangle_{1^{st}-\text{layer } TiO_2}$. Along the linear interpolation connecting the initial and final geometries, structures were sampled at 25, 30, 35, 50, and 75% of the path, and the CDFT energies of the initial charge-separated and final charge-recombined states were evaluated as functions of Δz. The $4Au^{+1.0e}/3TiO_2^{-1.0e}$ initial charge-separated state and the $4Au^{0.0e}/3TiO_2^{0.0e}$ final charge-recombined state are effectively degenerate at the 30% structure, which we identify as the transition state (TS) geometry (see **Figure S3** and **S4**). Moreover, **Figure S3a** presents the harmonic PES of the initial charge-separated state and the anharmonic PES of the final charge-recombined state. This visual observation is supported by the R^2 values of each PES with the initial charge-separated state containing a good harmonic fit ($R^2 = 0.99$) and the final charge-recombined state deviating from harmonicity ($R^2 = 0.51$). The fitted curvatures (force constants, k) are nevertheless comparable for the two PESs. We also note that Δz is a simplified and therefore approximate reaction coordinate, which is evident by the slight nonlinearity of the diabatic energy gap vs. Δz[30] in **Figure S3b**. Taken together, these results indicate that Marcus theory is appropriate to predict charge transfer trends across the Au/TiO_2 heterojunction due to the quasi-harmonic nature of the Au/TiO_2 system, but quantitative timescales should be viewed with caution.

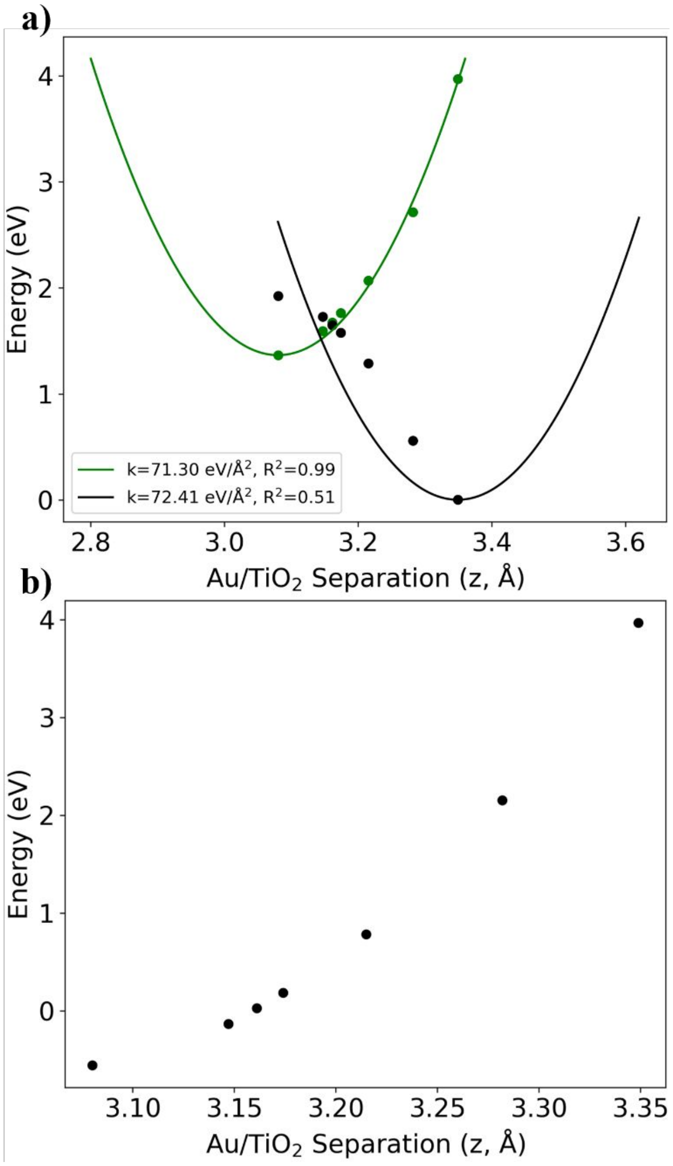

Figure S3. Diabatic potential energy surfaces (PESs) with respect to the vertical Au/TiO_2 separation ($\Delta z = \langle z \rangle_{4\text{Au}} - \langle z \rangle_{1^{\text{st}}-\text{layer TiO}_2}$). (a) PESs of the $4Au^{+1.0e}/3TiO_2^{-1.0e}$ initial charge-separated state and $4Au^{0.0e}/3TiO_2^{0.0e}$ final charge-recombined state vs. Δz. (b) Diabatic energy gap vs. Δz. The legend contains the curvature/force constant (k) and R^2 values for each PES. The transition state geometry Δz = 3.17 Å and can be visualized in **Figure S4c**.

Having determined the TS geometry and energy, we estimated the CDFT+TS theory (TST) charge recombination timescale. Here, the activation energy ($\Delta G^{\ddagger}$) is the TS energy minus the $4Au^{+1.0e}/3TiO_2^{-1.0e}$ initial charge-separated state energy in the initial geometry, yielding $\Delta G^{\ddagger}$ = 0.307 eV. This barrier is roughly twice the Marcus theory prediction of $\Delta G^{\ddagger} = \frac{(\lambda+\Delta G)^2}{4\lambda}$ = 0.147 eV. Using the same dominant vibrational frequency (υ_n) as the prefactor in adiabatic Marcus theory (see **eq 4** and **Section S7**), the CDFT+TST charge recombination timescale is 7.78 nanoseconds (ns). Conversely, the CDFT+Marcus theory charge recombination timescale is 15.95

ps (see **Section S7**), which is in closer agreement with the reported 60 ps experiment[33]. We therefore favor the CDFT+Marcus theory framework for quantitative timescales, as it better reproduces the experiment and does not require an explicit TS search.

S6: Analyses of Charge Recombination at the Initial and Final Geometries

The focus in the main paper (**Figures 1, 2** and **Tables 1**, **2**) is on analyzing charge recombination at the final geometry, which is due to the ease of visualizing charge transferring from the initial charge-separated state to the final charge-recombined state. For completeness, we recreate **Figure 1**, **Table 1**, **Figure 2**, and **Table 2**, with the inclusion of geometry change as **Figure S4**, **Table S5**, **Figure S5**, and **Table S6**, respectively. Once geometry change is included in the charge density map (plotted using VESTA[34]), the charge density difference (final charge-recombined state minus initial charge-separated state) is dominated by the atoms changing positions. **Figure S4a** demonstrates that charge density difference is due to atomic position change between the initial and final geometries. Here, it becomes visually unclear to determine where the charge is transferring to and from, counter to **Figure 1** at the final geometry. **Figure S4** also includes the initial, transition state, and final geometries with the index of each Au atom (see **Table 1**).

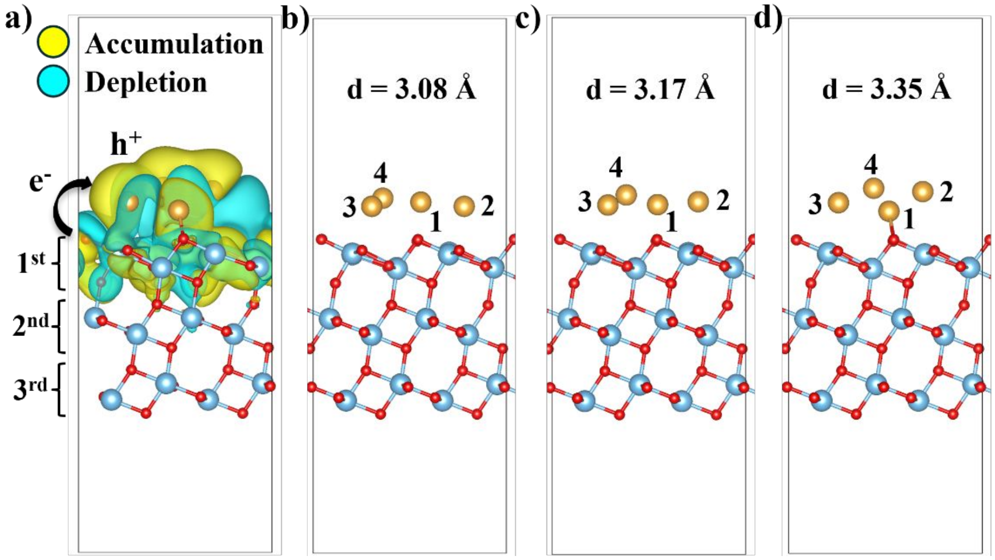

Figure S4. Charge density difference map and geometries of $4Au/3TiO_2$. (a) Charge density difference map, $4Au^{0.0e}/3TiO_2^{0.0e}$ final charge-recombined state at the final geometry minus $4Au^{+1.0e}/3TiO_2^{-1.0e}$ initial charge-separated state at the initial geometry, with a 0.001 e/Å^3 isovalue. The (b) $4Au^{+1.0e}/3TiO_2^{-1.0e}$ initial geometry, (c) transition state geometry, and (d) $4Au^{0.0e}/3TiO_2^{0.0e}$ final geometry. The Au/TiO_2 separation is defined as $\Delta z = \langle z \rangle_{4Au} - \langle z \rangle_{1^{st}-\text{layer } TiO_2}$.

Table S5 demonstrates that charge density depletion is predominantly localized in the 1st layer of $3TiO_2$ (like in **Table 1**) and is quantified through the +0.975 e difference between the final charge-recombined and initial charge-separated states at their respective geometries. Hirshfeld charge analysis reveals that charge recombination occurs at the surface of the 4Au cluster (see **Table S5** and **Figure S4b,c**). In particular, Au atom 4—furthest atom from $3TiO_2$ in the z direction—

acquires the least amount of charge (−0.185 *e*). By contrast, Au atom 1—closest atom to $3TiO_2$—acquires the largest amount of charge (−0.312 *e*). Furthermore, Au atoms 2 and 3, which lie at an intermediate distance from $3TiO_2$, gain a moderate and comparable quantity of charge (−0.256 and −0.244 *e*, respectively). After charge recombination, the 4Au cluster is polarized since Au atoms 1 and 2 flip their charges to negative while Au atoms 3 and 4 remain positive.

Table S5. Hirshfeld charges of the initial charge-separated and final charge-recombined states and their differences (final minus initial) at their respective geometries.

Hirshfeld (*e*)	$4Au^{+1.0e}/3TiO_2^{-1.0e}$	$4Au^{0.0e}/3TiO_2^{0.0e}$	Difference
Au atom 1	0.178	−0.134	−0.312
Au atom 2	0.236	−0.020	−0.256
Au atom 3	0.307	0.063	−0.244
Au atom 4	0.280	0.095	−0.185
4Au Cluster	1.001	0.004	−0.997
1st Layer of TiO_2	−0.572	0.403	0.975
2nd Layer of TiO_2	−0.426	−0.406	0.020
3rd Layer of TiO_2	0.001	0.002	0.001
$3TiO_2$	−0.997	−0.001	0.995

Next, we investigate the atom projected density of states (PDOS) plots and the highest occupied molecular orbital (HOMO) and lowest unoccupied molecular orbital (LUMO) charge density plots of the initial charge-separated state and final charge-recombined state at their respective geometries (see **Figure S5**). Here, **Figure S5b** is the same as **Figure 2b** meaning the analysis is the same as in the main paper. Thus, charge density is primarily localized on the 4Au cluster at the HOMO with minor charge density in the 1st layer of $3TiO_2$. For the LUMO, charge density is primarily delocalized in $3TiO_2$ with the addition of charge localized on 4Au, which is contrary to **Figure 2a** at the final geometry. The overlap in charge densities in the 1st layer of $3TiO_2$ and the 4Au cluster may be facilitating charge recombination from the LUMO to the HOMO.

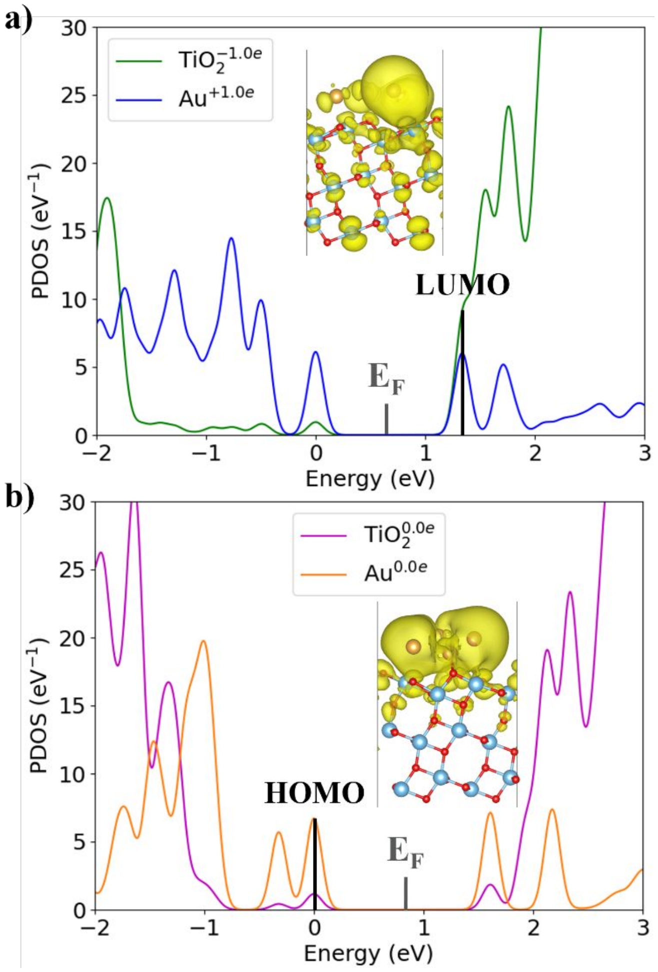

Figure S5. Visualization of charge density in the LUMO and HOMO and atom PDOS (aligned at the HOMO) in the 4Au/3TiO_2 system. (a) Atom PDOS and LUMO charge density plot of the 4$Au^{+1.0e}$/3$TiO_2^{-1.0e}$ initial charge-separated state at the initial geometry. (b) Atom PDOS and HOMO charge density plot of the 4$Au^{0.0e}$/3$TiO_2^{0.0e}$ final charge-recombined state at the final geometry. Both charge density plots use an isovalue of 0.001 e/Å^3.

According to the PDOS, an energy shift takes place in the LUMO that increases the HOMO−LUMO gap following charge recombination (see **Table S6**). This shift is attributed to the formation of a bipolaron with a binding energy of 0.277 eV, indicating strong electron-hole attraction (see **Figure S5** and **Table S6**). The observed band structure renormalization is consistent with the presence of polarons in anatase TiO_2[35,36]. Additionally, the V_c and dipole moment (p) both reduce enough to flip their directions between the initial charge-separated and final charge-recombined states.

Table S6. HOMO−LUMO gap, V_c, and dipole moment (p) of the initial charge-separated state at the initial geometry and final charge-recombined state at the final geometry and their differences (final minus initial).

Energy (eV)	4$Au^{+1.0e}$/3$TiO_2^{-1.0e}$	4$Au^{0.0e}$/3$TiO_2^{0.0e}$	Difference

HOMO−LUMO gap	1.333	1.610	0.277
V_c (4Au cluster)	1.740	−0.058	−1.798
p (e·Å) (z direction)	0.954	−0.172	−1.126

S7: Determining υ_n for Adiabatic Charge Recombination

In order to use **eq 4**, the υ_n governing charge recombination across the Au/TiO_2 heterojunction must be identified. Here, we conducted a 0 K DFT phonon mode ($\tilde{\upsilon}$) calculation of 4Au/3TiO_2 at the final charge-recombined state using GPAW with all atoms being relaxed. We find that the $\tilde{\upsilon}$ from 0 to 50 cm^{-1} in **Figure 3** are from Ti and O atoms. There are 10 Au atom $\tilde{\upsilon}$ between 50 and 200 cm^{-1} (see **Figure S6**). The $\tilde{\upsilon}$ above 200 cm^{-1} are primarily from O atom vibrations with minor contributions from Ti atom vibrations.

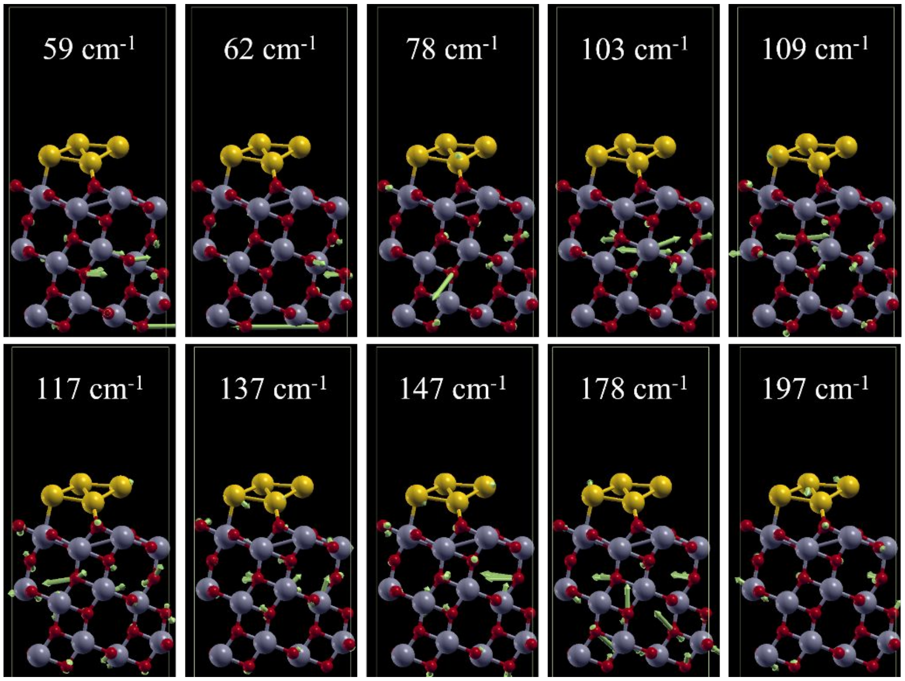

Figure S6. Au atom phonon modes ($\tilde{\upsilon}$) in the 4Au/3TiO_2 system at the final charge-recombined state and final geometry.

Charge recombination is likely dictated by the vibrational $\tilde{\upsilon}$ that follows the reaction coordinate (**Section S5**) and exhibits strong electron-phonon (e-ph) coupling. In **Section S5** we find that the vertical Au/TiO_2 separation (reaction coordinate) is smaller in the initial charge-separated state geometry than in the final charge-recombined state geometry. Accordingly, we present the mode vectors of 14 potential charge transfer modes from 359 to 931 cm^{-1} in **Figure S7**, obtained from a 0 K DFT frequency calculation at the final geometry using GPAW. In the 628 cm^{-1} mode the O atom bonded to Au (Au atom 1 in **Figure S4**) moves toward the Au atom and O atom mode vectors point upward toward the 4Au cluster. We therefore assign the 628 cm^{-1} $\tilde{\upsilon}$ as the most probable charge recombination mode and use its corresponding υ as υ_n in the main text (**Table 3** and **Table S7**). We then compare this selected υ_n with prior literature and with the HOMO−LUMO phonon

influence spectrum of the 4Au/3TiO_2 system from AIMD simulations. According to a DFT+U study in bulk anatase TiO_2, polar longitudinal-optical (LO) $\tilde{\upsilon}$ contain the largest e-ph couplings[36]. The two dominant e-ph coupling polar LO $\tilde{\upsilon}$ are at 343 and 874 cm^{-1}, with the latter dominating the electron scattering rate for polaron transport[36]. These $\tilde{\upsilon}$ are charge transport modes in TiO_2 and are not necessarily the charge transfer modes across the 4Au/3TiO_2 interface. In **Figure 3**, several high intensity peaks above 300 cm^{-1} are probable charge transfer modes. In our AIMD results the most intense peak above 300 cm^{-1} occurs at 650 cm^{-1} and no e-ph coupling is observed above 800 cm^{-1} (**Figure 3**). Overall, the 650 cm^{-1} peak agrees with our selected υ_n and shows strong e-ph coupling.

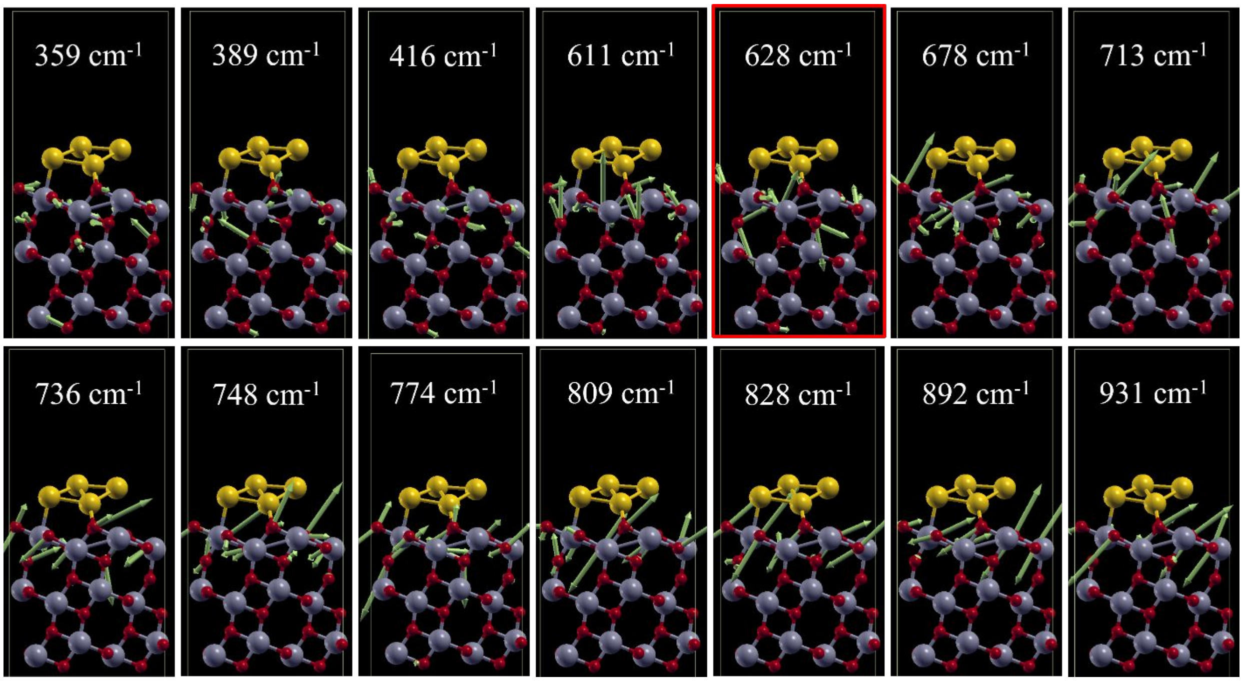

Figure S7. Potential charge recombination phonon modes ($\tilde{\upsilon}$) in the 4Au/3TiO_2 system at the final charge-recombined state and final geometry. The red square indicates the most probable $\tilde{\upsilon}$ to facilitate charge recombination.

Table S7. Adiabatic charge recombination timescales across the 4Au/3TiO_2 interface with respect to phonon modes ($\tilde{\upsilon}$) and their corresponding υ. Bold indicates the υ used as υ_n in the main paper.

$\tilde{\upsilon}$ (cm^{-1})	359	389	416	611	**628**	678	713	736	748	774	809	828	892	931
υ (THz)	10.76	11.66	12.47	18.32	**18.83**	20.33	21.38	22.07	22.42	23.20	24.25	24.82	26.74	27.91
κ_{el}	0.98	0.98	0.97	0.92	**0.92**	0.90	0.89	0.89	0.88	0.88	0.87	0.86	0.85	0.84
$1/k_{ET}^{ad}$ (ps)	27.90	25.75	24.08	16.40	**15.95**	14.78	14.05	13.61	13.39	12.94	12.38	12.10	11.23	10.76

S8: Electron-Hole Recombination with NAMD

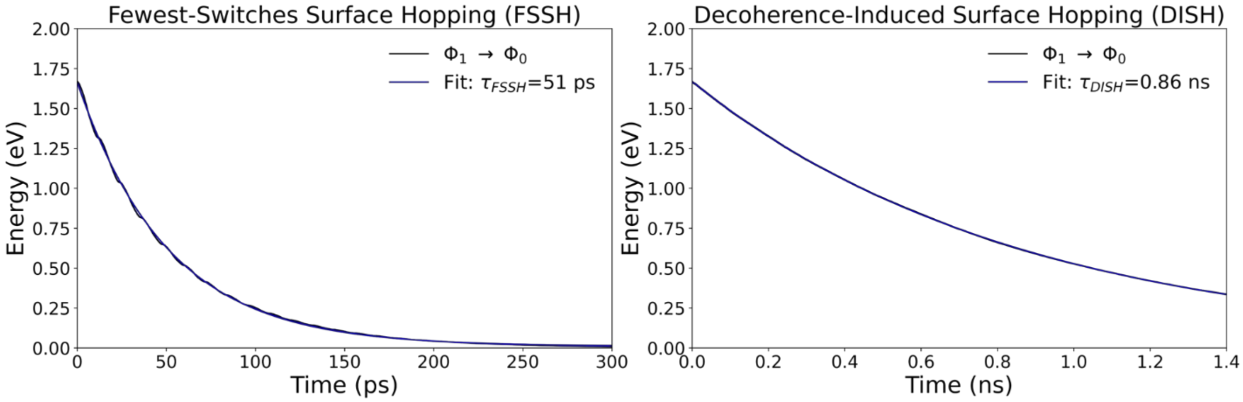

Figure S8. Energy decay of the excited state (Φ_1) electron population in the $4Au/3TiO_2$ system with FSSH (left) and DISH (right) at 300 K. The charge recombination lifetimes of FSSH and DISH are 51 and 860 ps, respectively.

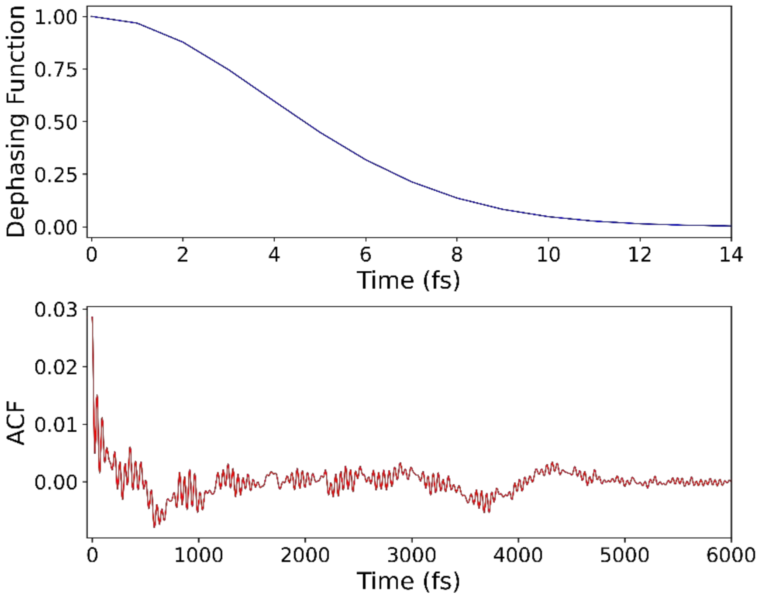

Figure S9. The dephasing function (top) and autocorrelation function (bottom) with respect to time in the VASP AIMD $4Au/3TiO_2$ systems at 300 K. The dephasing time is 4 femtoseconds (fs).

S9: Atom PDOS of the $5Au/3TiO_2$ System

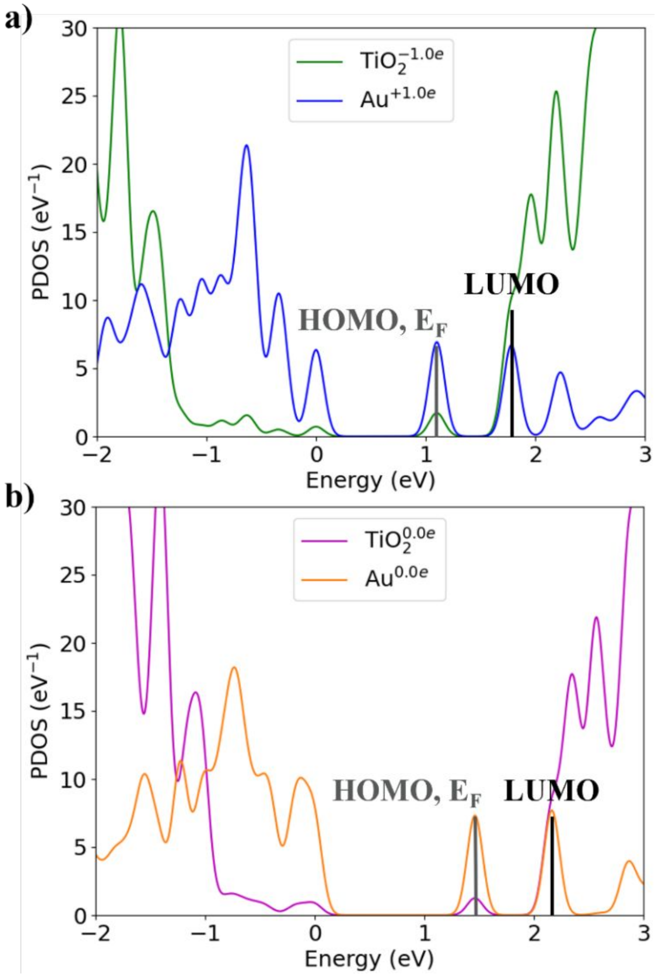

Figure S10. Visualization of the atom PDOS in the $5Au/3TiO_2$ system aligned at the HOMO-1. (a) Atom PDOS of the $4Au^{+1.0e}/3TiO_2^{-1.0e}$ initial charge-separated state at the initial geometry. (b) Atom PDOS of the $4Au^{0.0e}/3TiO_2^{0.0e}$ final charge-recombined state at the final geometry.

S10: GPAW Structures, Inputs, and Outputs

All of the GPAW structures, inputs, and output files are placed in Drew M. Glenna's GitHub (https://github.com/DrewGlenna/Site-Specific-Charge-Recombination-Dynamics-With-CDFT). A zip file is also included as a supplementary file.

S11: References

(1) Kresse, G.; Hafner, J. *Ab Initio* Molecular Dynamics for Liquid Metals. *Phys. Rev. B* **1993**, *47* (1), 558–561. https://doi.org/10.1103/PhysRevB.47.558.

(2) Kresse, G.; Furthmüller, J. Efficiency of Ab-Initio Total Energy Calculations for Metals and Semiconductors Using a Plane-Wave Basis Set. *Computational Materials Science* **1996**, *6* (1), 15–50. https://doi.org/10.1016/0927-0256(96)00008-0.

(3) Kresse, G.; Furthmüller, J. Efficient Iterative Schemes for *Ab Initio* Total-Energy Calculations Using a Plane-Wave Basis Set. *Phys. Rev. B* **1996**, *54* (16), 11169–11186. https://doi.org/10.1103/PhysRevB.54.11169.

(4) Kresse, G.; Joubert, D. From Ultrasoft Pseudopotentials to the Projector Augmented-Wave Method. *Phys. Rev. B* **1999**, *59* (3), 1758–1775. https://doi.org/10.1103/PhysRevB.59.1758.
(5) Mortensen, J. J.; Hansen, L. B.; Jacobsen, K. W. Real-Space Grid Implementation of the Projector Augmented Wave Method. Phys. Rev. B 2005, 71 (3), 035109. https://doi.org/10.1103/PhysRevB.71.035109.
(6) Enkovaara, J.; Rostgaard, C.; Mortensen, J. J.; Chen, J.; Dułak, M.; Ferrighi, L.; Gavnholt, J.; Glinsvad, C.; Haikola, V.; Hansen, H. A.; Kristoffersen, H. H.; Kuisma, M.; Larsen, A. H.; Lehtovaara, L.; Ljungberg, M.; Lopez-Acevedo, O.; Moses, P. G.; Ojanen, J.; Olsen, T.; Petzold, V.; Romero, N. A.; Stausholm-Møller, J.; Strange, M.; Tritsaris, G. A.; Vanin, M.; Walter, M.; Hammer, B.; Häkkinen, H.; Madsen, G. K. H.; Nieminen, R. M.; Nørskov, J. K.; Puska, M.; Rantala, T. T.; Schiøtz, J.; Thygesen, K. S.; Jacobsen, K. W. Electronic Structure Calculations with GPAW: A Real-Space Implementation of the Projector Augmented-Wave Method. J. Phys. Condens. Matter 2010, 22 (25), 253202. https://doi.org/10.1088/0953-8984/22/25/253202.
(7) Mortensen, J. J.; Larsen, A. H.; Kuisma, M.; Ivanov, A. V.; Taghizadeh, A.; Peterson, A.; Haldar, A.; Dohn, A. O.; Schäfer, C.; Jónsson, E. Ö.; Hermes, E. D.; Nilsson, F. A.; Kastlunger, G.; Levi, G.; Jónsson, H.; Häkkinen, H.; Fojt, J.; Kangsabanik, J.; Sødequist, J.; Lehtomäki, J.; Heske, J.; Enkovaara, J.; Winther, K. T.; Dulak, M.; Melander, M. M.; Ovesen, M.; Louhivuori, M.; Walter, M.; Gjerding, M.; Lopez-Acevedo, O.; Erhart, P.; Warmbier, R.; Würdemann, R.; Kaappa, S.; Latini, S.; Boland, T. M.; Bligaard, T.; Skovhus, T.; Susi, T.; Maxson, T.; Rossi, T.; Chen, X.; Schmerwitz, Y. L. A.; Schiøtz, J.; Olsen, T.; Jacobsen, K. W.; Thygesen, K. S. GPAW: An Open Python Package for Electronic Structure Calculations. J. Chem. Phys. 2024, 160 (9), 092503. https://doi.org/10.1063/5.0182685.
(8) Melander, M.; Jónsson, E. Ö.; Mortensen, J. J.; Vegge, T.; García Lastra, J. M. Implementation of Constrained DFT for Computing Charge Transfer Rates within the Projector Augmented Wave Method. *J. Chem. Theory Comput.* **2016**, *12* (11), 5367–5378. https://doi.org/10.1021/acs.jctc.6b00815.
(9) Kaduk, B.; Kowalczyk, T.; Van Voorhis, T. Constrained Density Functional Theory. Chem. Rev. 2012, 112 (1), 321–370. https://doi.org/10.1021/cr200148b.
(10) Marcus, R. A. On the Theory of Oxidation-Reduction Reactions Involving Electron Transfer. I. J. Chem. Phys. 1956, 24 (5), 966–978. https://doi.org/10.1063/1.1742723.
(11) Hjorth Larsen, A.; Jørgen Mortensen, J.; Blomqvist, J.; Castelli, I. E.; Christensen, R.; Dułak, M.; Friis, J.; Groves, M. N.; Hammer, B.; Hargus, C.; Hermes, E. D.; Jennings, P. C.; Bjerre Jensen, P.; Kermode, J.; Kitchin, J. R.; Leonhard Kolsbjerg, E.; Kubal, J.; Kaasbjerg, K.; Lysgaard, S.; Bergmann Maronsson, J.; Maxson, T.; Olsen, T.; Pastewka, L.; Peterson, A.; Rostgaard, C.; Schiøtz, J.; Schütt, O.; Strange, M.; Thygesen, K. S.; Vegge, T.; Vilhelmsen, L.; Walter, M.; Zeng, Z.; Jacobsen, K. W. The Atomic Simulation Environment—a Python Library for Working with Atoms. *J. Phys.: Condens. Matter* **2017**, *29* (27), 273002. https://doi.org/10.1088/1361-648X/aa680e.
(12) Hummer, D. R.; Heaney, P. J.; Post, J. E. Thermal Expansion of Anatase and Rutile between 300 and 575 K Using Synchrotron Powder X-Ray Diffraction. Powder Diffr. 2007, 22 (4), 352–357. https://doi.org/10.1154/1.2790965.
(13) Grimme, S.; Antony, J.; Ehrlich, S.; Krieg, H. A Consistent and Accurate *Ab Initio* Parametrization of Density Functional Dispersion Correction (DFT-D) for the 94 Elements H-Pu. *The Journal of Chemical Physics* **2010**, *132* (15), 154104. https://doi.org/10.1063/1.3382344.

(14) Blöchl, P. E. Projector Augmented-Wave Method. *Phys. Rev. B* **1994**, *50* (24), 17953–17979. https://doi.org/10.1103/PhysRevB.50.17953.
(15) Perdew, J. P.; Burke, K.; Ernzerhof, M. Generalized Gradient Approximation Made Simple. *Phys. Rev. Lett.* **1996**, *77* (18), 3865–3868. https://doi.org/10.1103/PhysRevLett.77.3865.
(16) Henkelman, G.; Arnaldsson, A.; Jónsson, H. A Fast and Robust Algorithm for Bader Decomposition of Charge Density. Comput. Mater. Sci. 2006, 36 (3), 354–360. https://doi.org/10.1016/j.commatsci.2005.04.010.
(17) Sanville, E.; Kenny, S. D.; Smith, R.; Henkelman, G. Improved Grid-Based Algorithm for Bader Charge Allocation. J. Comput. Chem. 2007, 28 (5), 899–908. https://doi.org/10.1002/jcc.20575.
(18) Tang, W.; Sanville, E.; Henkelman, G. A Grid-Based Bader Analysis Algorithm without Lattice Bias. J. Phys. Condens. Matter 2009, 21 (8), 084204. https://doi.org/10.1088/0953-8984/21/8/084204.
(19) Yu, M.; Trinkle, D. R. Accurate and Efficient Algorithm for Bader Charge Integration. J. Chem. Phys. 2011, 134 (6), 064111. https://doi.org/10.1063/1.3553716.
(20) Hirshfeld, F. L. Bonded-Atom Fragments for Describing Molecular Charge Densities. Theor. Chim. Acta 1977, 44 (2), 129–138. https://doi.org/10.1007/BF00549096.
(21) Anisimov, V. I.; Zaanen, J.; Andersen, O. K. Band Theory and Mott Insulators: Hubbard U Instead of Stoner I. Phys. Rev. B 1991, 44 (3), 943–954. https://doi.org/10.1103/PhysRevB.44.943.
(22) Dudarev, S. L.; Botton, G. A.; Savrasov, S. Y.; Humphreys, C. J.; Sutton, A. P. Electron-Energy-Loss Spectra and the Structural Stability of Nickel Oxide: An LSDA+U Study. Phys. Rev. B 1998, 57 (3), 1505–1509. https://doi.org/10.1103/PhysRevB.57.1505.
(23) Tang, H.; Prasad, K.; Sanjinès, R.; Schmid, P. E.; Lévy, F. Electrical and Optical Properties of TiO_2 Anatase Thin Films. *J. Appl. Phys.* **1994**, *75* (4), 2042–2047. https://doi.org/10.1063/1.356306.
(24) Gumber, S.; Prezhdo, O. V. Energy-Conserving Surface Hopping for Auger Processes. *J. Chem. Theory Comput.* **2024**, *20* (13), 5408–5417. https://doi.org/10.1021/acs.jctc.4c00562.
(25) Jaeger, H. M.; Fischer, S.; Prezhdo, O. V. Decoherence-Induced Surface Hopping. *The Journal of Chemical Physics* **2012**, *137* (22), 22A545. https://doi.org/10.1063/1.4757100.
(26) Akimov, A. V.; Prezhdo, O. V. Persistent Electronic Coherence Despite Rapid Loss of Electron–Nuclear Correlation. J. Phys. Chem. Lett. 2013, 4 (22), 3857–3864. https://doi.org/10.1021/jz402035z.
(27) Long, R.; Prezhdo, O. V. Instantaneous Generation of Charge-Separated State on TiO_2 Surface Sensitized with Plasmonic Nanoparticles. *J. Am. Chem. Soc.* **2014**, *136* (11), 4343–4354. https://doi.org/10.1021/ja5001592.
(28) Akimov, A. V. Libra: An Open-Source "Methodology Discovery" Library for Quantum and Classical Dynamics Simulations: SOFTWARE NEWS AND UPDATES. J. Comput. Chem. 2016, 37 (17), 1626–1649. https://doi.org/10.1002/jcc.24367.
(29) Migliore, A. Nonorthogonality Problem and Effective Electronic Coupling Calculation: Application to Charge Transfer in π-Stacks Relevant to Biochemistry and Molecular Electronics. J. Chem. Theory Comput. 2011, 7 (6), 1712–1725. https://doi.org/10.1021/ct200192d.
(30) Wu, Q.; Van Voorhis, T. Extracting Electron Transfer Coupling Elements from Constrained Density Functional Theory. J. Chem. Phys. 2006, 125 (16), 164105. https://doi.org/10.1063/1.2360263.

(31) Wang, Y.-C.; Jiang, H. Constrained Density Functional Theory plus the Hubbard U Correction Approach for the Electronic Polaron Mobility: A Case Study of TiO_2. Chin. J. Chem. Phys. 2021, 34 (5), 541–551. https://doi.org/10.1063/1674-0068/cjcp2108136.
(32) Wu, Q.; Van Voorhis, T. Constrained Density Functional Theory and Its Application in Long-Range Electron Transfer. J. Chem. Theory Comput. 2006, 2 (3), 765–774. https://doi.org/10.1021/ct0503163.
(33) Borgwardt, M.; Mahl, J.; Roth, F.; Wenthaus, L.; Brauße, F.; Blum, M.; Schwarzburg, K.; Liu, G.; Toma, F. M.; Gessner, O. Photoinduced Charge Carrier Dynamics and Electron Injection Efficiencies in Au Nanoparticle-Sensitized TiO_2 Determined with Picosecond Time-Resolved X-Ray Photoelectron Spectroscopy. *J. Phys. Chem. Lett.* **2020**, *11* (14), 5476–5481. https://doi.org/10.1021/acs.jpclett.0c00825.
(34) Momma, K.; Izumi, F. VESTA 3 for Three-Dimensional Visualization of Crystal, Volumetric and Morphology Data. J. Appl. Crystallogr. 2011, 44, 1272–1276. https://doi.org/10.1107/S0021889811038970.
(35) Mou, T.; Gupta, V. K.; Tabriz, M. F.; Frauenheim, T.; Deák, P. Size of Electron Polarons in Anatase TiO_2 and Their Role in Photocatalysis. Phys. Rev. B 2023, 107 (15), 155127. https://doi.org/10.1103/PhysRevB.107.155127.
(36) Kang, Y.; Peelaers, H.; Van De Walle, C. G. First-Principles Study of Electron-Phonon Interactions and Transport in Anatase TiO_2. Phys. Rev. B 2019, 100 (12), 121113. https://doi.org/10.1103/PhysRevB.100.121113.
