# Supplementary material for: Real-Space Constrained Density Functional Theory Investigation of Site-Specific, Interfacial Charge Recombination Dynamics Across the Au Nanoparticle/TiO2 Heterojunction
Source: J Phys Chem Lett. 2026 Jan 6;17(2):570–8. doi: 10.1021/acs.jpclett.5c02905 (PMC12814526; doi:10.1021/acs.jpclett.5c02905)
Supplement: Supplementary file 2 [file jz5c02905_si_002.pdf]

jz-2025-02905b.R1

Name: Peer Review Information for "Real-Space Constrained DFT Investigation of Site-Specific, Interfacial Charge Recombination Dynamics Across the Au Nanoparticle/TiO2 Heterojunction"

First Round of Reviewer Comments

Reviewer: 1

Comments to the Author

This article uses Au/TiO2 as a prominent model system and discussed the applicability of a novel method constrained real-space DFT to study interfacial charge transfer. The pros and cons are benchmarked and discussed in conjunction with the more established, but also more costly NAMD methods. The depth of the article is good and the topic seems to be a good fit for JPCL.

With the promises of constrained real-space DFT being obvious in terms of computational cost and site-specific, I especially appreciate the honest discussion of potential limitations and pitfalls, namely, the exclusion of hybrid functionals and anharmonicity of the PES.

It would be useful if the authors can compare the simulation results with experiments. Also, can the method include long-wavelength phonon whose wavelength is larger than the supercell size?

Author's Response to Peer Review Comments:

We thank the editor and reviewer for their consideration and suggestions!

Below is our point by point response to the reviewer's comments. We are aware that this will not go back to the reviewer, but still appreciate their comment which helps us make a couple more clarifications/edits in the manuscript.

1. It would be useful if the authors can compare the simulation results with experiments.

We thank the reviewer for this helpful suggestion. As noted in the Introduction (third paragraph on page 2), constrained DFT (CDFT) is "site-specific in its buildup, which in principle, reflects the physical picture measured by time-resolved X-ray Photoelectron Spectroscopy (trXPS) experiment (Borgwardt, M.; Mahl, J.; Roth, F.; Wenthaus, L.; Brauße, F.; Blum, M.; Schwarzburg, K.; Liu, G.; Toma, F. M.; Gessner, O. Photoinduced Charge Carrier Dynamics and Electron Injection Efficiencies in Au Nanoparticle-Sensitized TiO2 Determined with Picosecond Time-Resolved X-Ray Photoelectron Spectroscopy. J. Phys. Chem. Lett. 2020, 11 (14), 5476–5481. https://doi.org/10.1021/acs.jpclett.0c00825)." This site specificity naturally motivates a comparison between our CDFT+Marcus theory charge recombination timescale and previously reported trXPS measurements.

In the revised manuscript, we have incorporated this discussion into the main text between Figures 4 and 5. Specifically, we added the following sentences to the second paragraph on page 12: "In addition, the 16 ps timescale for 1/k_ET^ad obtained from site-specific CDFT+Marcus theory is comparable to the previously reported recombination timescale of 60 ps from site-resolved trXPS measurements (Borgwardt, M.; Mahl, J.; Roth, F.; Wenthaus, L.; Brauße, F.; Blum, M.; Schwarzburg, K.; Liu, G.; Toma, F. M.; Gessner, O. Photoinduced Charge Carrier Dynamics and Electron Injection Efficiencies in Au Nanoparticle-Sensitized TiO2 Determined with Picosecond Time-Resolved X-Ray Photoelectron Spectroscopy. J. Phys. Chem. Lett. 2020, 11 (14), 5476–5481. https://doi.org/10.1021/acs.jpclett.0c00825). However, this apparent quantitative agreement should be interpreted with caution, as the trXPS experiments were performed on substantially larger Au/TiO2 interfaces, containing on the order of 2 × 105 Au atoms per NP (Borgwardt, M.; Mahl, J.; Roth, F.; Wenthaus, L.; Brauße, F.; Blum, M.; Schwarzburg, K.; Liu, G.; Toma, F. M.; Gessner, O. Photoinduced Charge Carrier Dynamics and Electron Injection Efficiencies in Au Nanoparticle-Sensitized TiO2 Determined with Picosecond Time-Resolved X-Ray Photoelectron Spectroscopy. J. Phys. Chem. Lett. 2020, 11 (14), 5476–5481. https://doi.org/10.1021/acs.jpclett.0c00825)." These sentences were inserted between "Notably, CDFT+Marcus theory recovers the NAMD recombination lifetime trends in the 4Au/3TiO2 system while demanding roughly 80% fewer node-hours and requires much less data storage than the NAMD simulations"

and “Therefore, the present CDFT+Marcus theory approach is sufficient to predict charge transfer trends across the Au/TiO2 heterojunction and at a fraction of the computational cost when compared to NAMD.”

2. Also, can the method include long-wavelength phonon whose wavelength is larger than the supercell size?

We thank the reviewer for this helpful comment. GPAW uses the finite-displacement method to calculate vibrational modes. In this approach, only phonons whose spatial periodicity is comparable to or smaller than the simulation cell are represented explicitly, while modes with wavelengths larger than the supercell are not captured (Giustino, F. Electron-Phonon Interactions from First Principles. Rev. Mod. Phys. 2017, 89 (1), 015003. https://doi.org/10.1103/RevModPhys.89.015003). As discussed by Giustino, long-wavelength modes can instead be modeled within reciprocal-space density functional perturbation theory (DFPT) (Baroni, S.; De Gironcoli, S.; Dal Corso, A.; Giannozzi, P. Phonons and Related Crystal Properties from Density-Functional Perturbation Theory. Rev. Mod. Phys. 2001, 73 (2), 515–562. https://doi.org/10.1103/RevModPhys.73.515), which computes phonon and electron–phonon properties at arbitrary wavevector q within the primitive cell and avoids the need for macroscopically large supercells.

We include this discussion in paragraph 2 on page 14 (limitations section) of our main manuscript. We state “In addition, the vibrational modes entering eq 4 were obtained from finite-displacement calculations using GPAW, which only capture phonons whose wavelengths are comparable to or smaller than the supercell (Giustino, F. Electron-Phonon Interactions from First Principles. Rev. Mod. Phys. 2017, 89 (1), 015003. https://doi.org/10.1103/RevModPhys.89.015003). Long-wavelength phonons can be treated using reciprocal-space density functional perturbation theory (DFPT) (Giustino, F. Electron-Phonon Interactions from First Principles. Rev. Mod. Phys. 2017, 89 (1), 015003. https://doi.org/10.1103/RevModPhys.89.015003; Baroni, S.; De Gironcoli, S.; Dal Corso, A.; Giannozzi, P. Phonons and Related Crystal Properties from Density-Functional Perturbation Theory. Rev. Mod. Phys. 2001, 73 (2), 515–562. https://doi.org/10.1103/RevModPhys.73.515). To our knowledge, no currently available code combines DFPT with CDFT, but the potential-based CDFT framework (Gonze, X.; Seddon, B.; Elliott, J. A.; Tantardini, C.; Shapeev, A. V. Constrained Density Functional Theory: A Potential-Based Self-Consistency Approach. J. Chem. Theory Comput. 2022, 18

(10), 6099–6110. https://doi.org/10.1021/acs.jctc.2c00673) is a natural route for such an extension."

We placed this discussion between the sentences "Here we calibrate the CDFT constraints (Section S3) and apply a Hubbard U correction (Section S4), which partially reduces H_ab, but our current computational resources forbid us from adopting a full hybrid-functional calculation" and "For Marcus theory, the assumption of harmonic PESs is most reliable for simple molecules (Marcus, R. A. On the Theory of Oxidation-Reduction Reactions Involving Electron Transfer. I. The Journal of Chemical Physics 1956, 24 (5), 966–978. https://doi.org/10.1063/1.1742723)." Lastly, coupling DFPT with CDFT to account for long-wavelength phonons at heterogeneous interfaces would be an interesting direction for future work, but lies beyond the scope of the present study.
